# The crystal structures of three clozapinium salts: different mol­ecular configurations, and supra­molecular assembly in one, two and three dimensions

**DOI:** 10.1107/S205698901500554X

**Published:** 2015-03-25

**Authors:** Manpreet Kaur, Jerry P. Jasinski, Hemmige S. Yathirajan, Channappa N. Kavitha, Christopher Glidewell

**Affiliations:** a Department of Studies in Chemistry, University of Mysore, Manasagangotri, Mysore 570 006, India; b Department of Chemistry, Keene State College, 229 Main Street, Keene, NH 03435-2001, USA; c School of Chemistry, University of St Andrews, St Andrews, Fife KY16 9ST, Scotland

**Keywords:** crystal structure, clozapinium, crystal structure, mol­ecular configuration, hydrogen bonding, supra­molecular assembly

## Abstract

The structures of three salts derived from clozapine, 8-chloro-11-(4-methyl­piperazin-1-yl)-5*H*-dibenzo[*b*,*e*][1,4]diazepine, are reported, namely, clo­za­pin­ium 3,5-di­nitro­benzoate dimethyl sulfoxide monosolvate, C_18_H_20_ClN_4_^+^·C_7_H_3_N_2_O_6_^−^·C_2_H_6_OS, (I), where the dimethyl sulfoxide component is disordered over two sets of atomic sites having occupancies 0.627 (2) and 0.373 (2); clo­za­pin­ium hydrogen maleate 0.21-hydrate, C_18_H_20_ClN_4_^+^·C_4_H_3_O_4_^−^·0.21H_2_O, (II), and clozapinium 2-hy­droxy­benzoate, C_18_H_20_ClN_4_^+^·C_7_H_5_O_3_^−^, (III). In all three salts, the protonation site is the methyl­ated N atom of the piperazine ring, and the dimensions and conformations of the fused tricyclic system are very similar. However, differences are apparent in the piperazine component: in both compounds (II) and (III), the unprotonated N atom of this ring has a pyramidal geometry, but in compound (I) this atom has a planar geometry. In compound (III), both N-substituents in this ring occupy equatorial sites, but in compound (II) the fused tricyclic system occupies an axial site of the piperazine ring. The independent components of compound (I) are linked within the selected asymmetric unit by a combination of N—H⋯O and C—H⋯O hydrogen bonds, and these hydrogen-bonded aggregates are linked into chains by an aromatic π–π stacking inter­action. In compound (II), the components are linked into sheets by a combination of O—H⋯O, N—H⋯O and C—H⋯O hydrogen bonds, and in compound (III), a combination of N—H⋯O, C—H⋯O and C—H⋯N hydrogen bonds links the components into a three-dimensional framework structure. Comparisons are made with some similar compounds.

## Abstract 2

In three simple clozapinium salts, the unprotonated N atom of the piperazine ring has been variously found to have a pyramidal or planar geometry, with the tricyclic component occupying either the axial or the equatorial position. The supra­molecular assembly is one-dimensional in the 3,5-di­nitro­benzoate salt, two-dimensional in the hydrogen maleate salt and three-dimensional in the 2-hy­droxy­benzoate salt.

## Chemical context   

**Figure 1:**
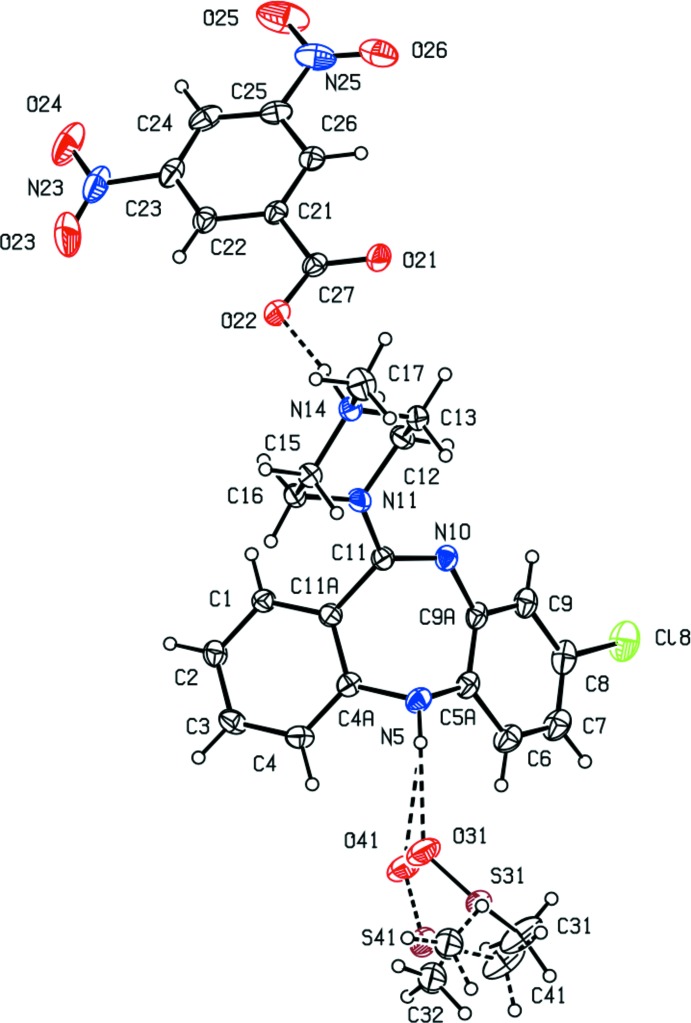
The independent components of compound (I), showing the atom-labelling scheme and the N—H⋯O hydrogen bonds within the selected asymmetric unit. Displacement ellipsoids are drawn at the 30% probability level and the major and minor orientations of the disordered dimethyl sulfoxide component, containing atoms S31 and S41, respectively, have occupancies 0.627 (2) and 0.373 (2).

**Figure 2:**
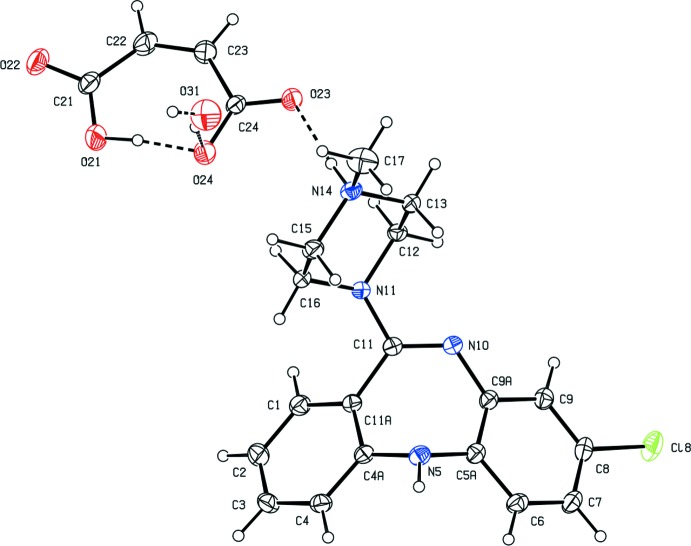
The independent components of compound (II), showing the atom-labelling scheme, and the O—H⋯O and N—H⋯O hydrogen bonds within the selected asymmetric unit. Displacement ellipsoids are drawn at the 30% probability level and the water mol­ecule, containing atom O31, has occupancy 0.210 (7).

**Figure 3:**
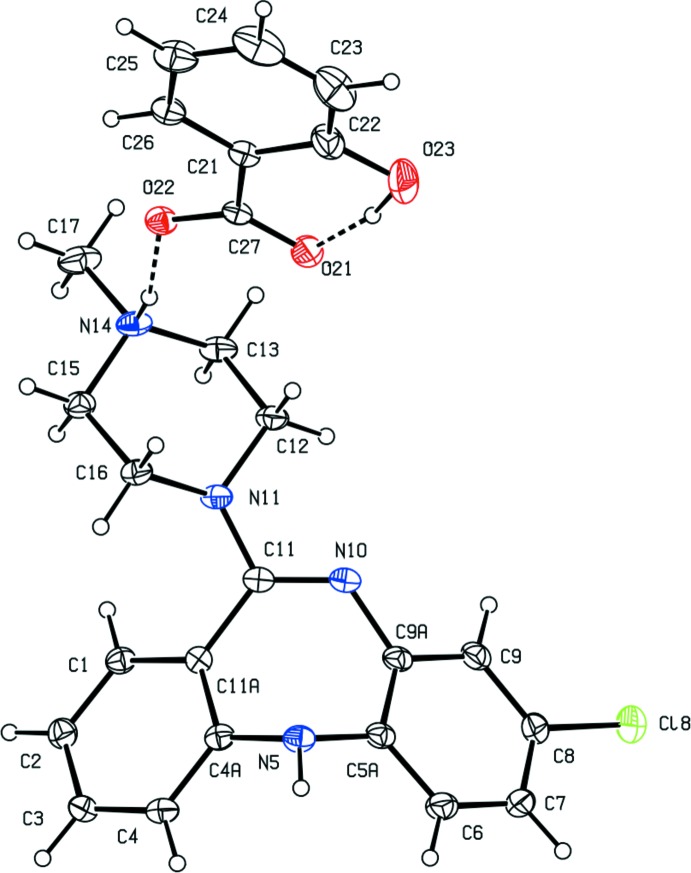
The independent components of compound (III), showing the atom-labelling scheme, and the O—H⋯O and N—H⋯O hydrogen bonds within the selected asymmetric unit. Displacement ellipsoids are drawn at the 30% probability level.

Clozapine, 8-chloro-11-(4-methyl­piperazin-1-yl)-5*H*-dibenzo[*b*,*e*][1,4]diazepine, is a well established medication used in the treatment of schizophrenia which in general leads to a lower incidence of adverse side effects such as Parkinsonian-type symptoms than some other treatments (Breier *et al.*, 1994 ▸). Although structures have been reported for both the free base itself (Petcher & Weber, 1976 ▸) and for its doubly-protonated di-cation, as the dibromide salt (Fillers & Hawkinson, 1982 ▸), there appear to be no reports of the structures of any monoprotonated clozapine derivatives. Accordingly, we have now determined the structures of three such salts with a variety of counter-ions. Of these salts, the 3,5-di­nitro­benzoate crystallizes from dimethyl sulfoxide as a stoichiometric monosolvate (I) (Fig. 1 ▸, Scheme 1); however, the hydrogen maleate crystallizes from the same solvent as a partial hydrate (II) (Fig. 2 ▸); and the 2-hy­droxy­benzoate crystallizes from a 1:1 mixture of aceto­nitrile and methanol in a solvent-free form (III) (Fig. 3 ▸). A number of other such salts were prepared, but no crystals suitable for single-crystal X-ray diffraction have so far been obtained from these, despite attempts to prepare crystals using a range of solvents. The aims of the present study are firstly to establish the site of protonation in the mono-protonated cations; secondly, to compare the conformations of the clozapinium cations; and thirdly, to explore the supra­molecular assembly in these three salts.
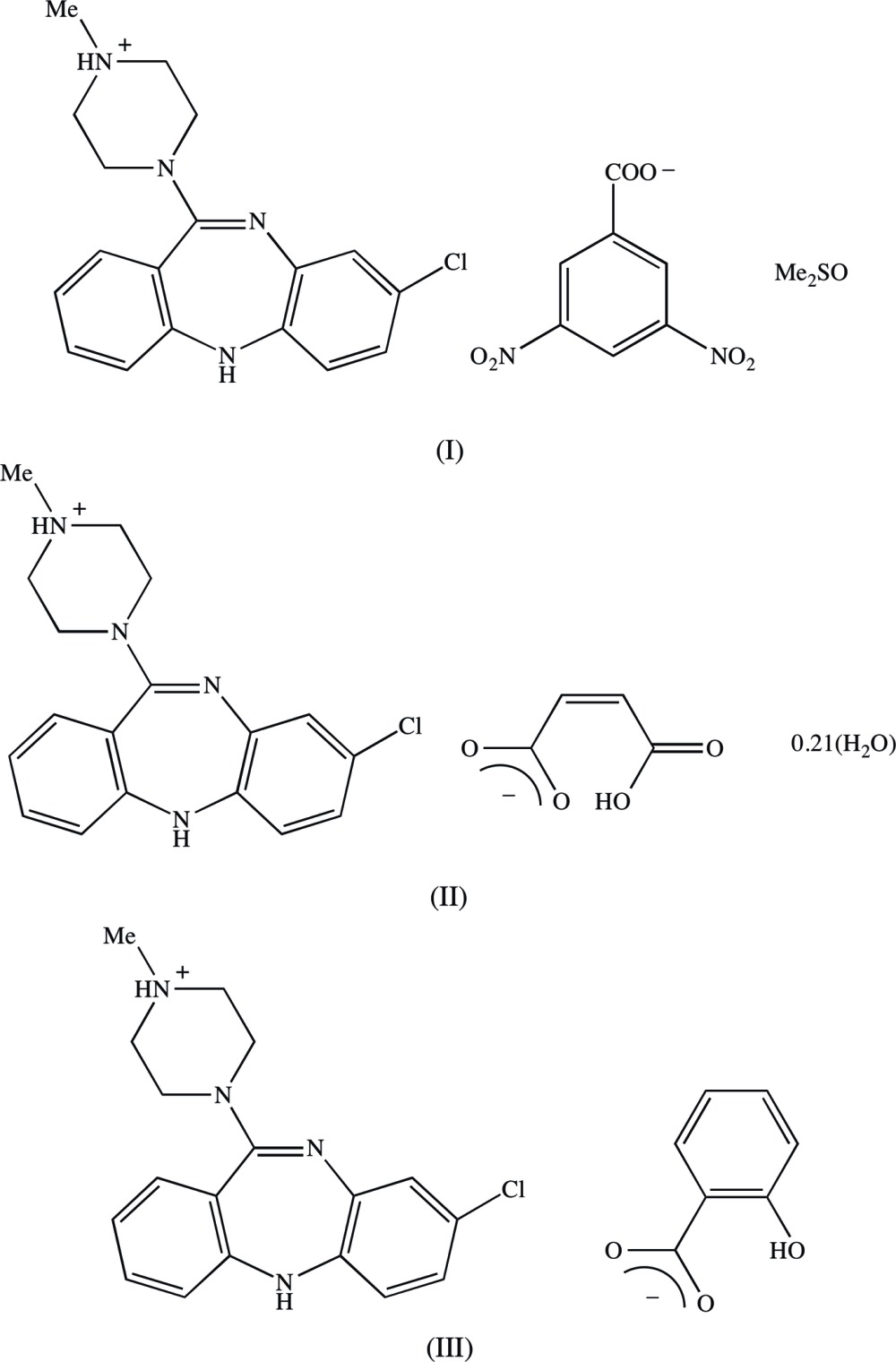


## Structural commentary   

Compound (I) consists of a clozapinium cation, in which protonation has occurred at the protonated N atom of the piperazine ring, as is also observed in both of compounds (II) and (III), a 3,5-di­nitro­benzoate anion, and a mol­ecule of dimethyl sulfoxide (DMSO), which is disordered over two orientations having site occupancies of 0.627 (2) and 0.273 (2), respectively (Fig. 1 ▸). It was possible to select an asymmetric unit for (I) in which the three components are linked by N—H⋯O hydrogen bonds (Fig. 1 ▸, Table 1 ▸). The N—H⋯O hydrogen bond between the two ionic components within the selected asymmetric unit is a charge-assisted hydrogen bond (Gilli *et al.*, 1994 ▸) and it is nearly linear with fairly short H⋯O and N⋯O distances (Table 1 ▸). There are also some short C—H⋯O contacts between the cation and the disordered DMSO components, but those to the major component, in particular, have long H⋯O distances, and C—H⋯O angles which are less than 140° (*cf.* Wood *et al.*, 2009 ▸) so these may be better regarded as adventitious contacts rather than structurally significant inter­actions.

Compound (II) consists of a clozapinium cation, a hydrogen maleate anion which contains a short intra-anion O—H⋯O hydrogen bond (Table 1 ▸), and a partial occupancy water mol­ecule whose refined site occupancy is 0.210 (7): despite the short O⋯O distance in the intra-anion hydrogen bond, the H atom is significantly displaced from a position equidistant from the two O atoms involved (Table 1 ▸). The two ionic components are linked by a nearly linear charge-assisted hydrogen bond (Fig. 2 ▸), while the partially occupied water site forms hydrogen bonds to two anions (Table 1 ▸).

There is again an intra-anion O—H⋯O hydrogen bond in the 2-hy­droxy­benzoate component of compound (III) and, again, the two ionic components are linked by a fairly short, charge-assisted hydrogen bond (Fig. 3 ▸). It is of inter­est to note both the general similarity in the dimensions of the two intra-anion hydrogen bonds in compounds (II) and (III), and also that in each of (I)–(III), the site of the protonation of the clozapine is the methyl­ated atom N14 of the piperazine ring. In each case, this N—H bond participates in a short charge-assisted hydrogen bond between the ionic components. As discussed below, this is the only N—H⋯O hydrogen bond involving the ionic components in both compound (I) and compound (III).

In the clozapinium cations of compounds (I)–(III), the fused tricyclic units exhibit very similar conformations, as shown by the relevant torsional and dihedral angles (Table 2 ▸) which define the relative orientations of the three rings. It is inter­esting to note that corresponding pairs of torsional angles involving either one or the other of the two aryl rings consistently have similar magnitudes but opposite signs, indicative of near mirror symmetry, provided the difference in the atomic types N10 and C11 is ignored, with the pseudo mirror containing the bond N5—H5 and passing through the mid-point of the bond N10—C11. However, there are some inter­esting differences between (I)–(III) in respect of the piperazine rings, which in each compound adopt a chair conformation, with protonation at the methyl­ated atom N14, where the methyl atom C17 always occupies the equatorial site. While the geometry at N11 is planar within experimental uncertainty in compound (I), it is pyramidal in each of (II) and (III). In addition, the atom C11 (and hence the bulky tricyclic system) occupies the equatorial site at N11 in compound (III), but in compound (II) the tricyclic unit unexpectedly occupies the axial site at atom N11, as indicated by the values of the torsional angles C11—N11—C12—C13 for these two compounds (Table 2 ▸).

## Supra­molecular features   

**Figure 4:**
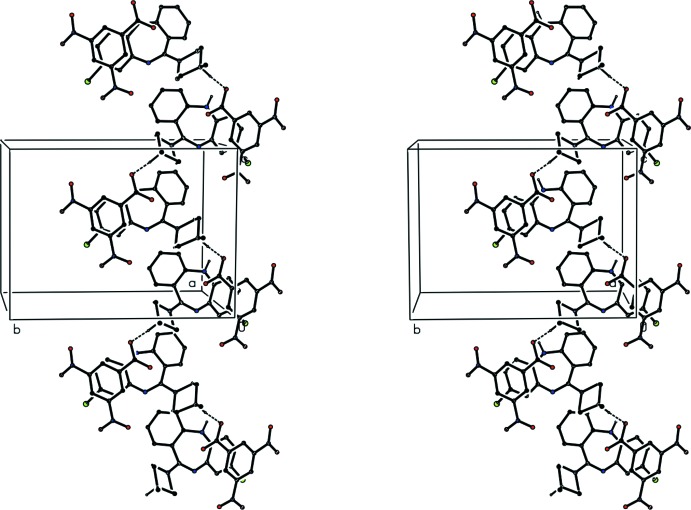
A stereoview of part of the crystal structure of compound (I), showing the formation of a π-stacked chain of hydrogen-bonded ion pairs. For the sake of clarity, H atoms not involved in the hydrogen bonds (shown as dashed lines) have been omitted, as have the disordered DMSO mol­ecules.

The supra­molecular assembly in compounds (I)–(III) provides structures in one, two and three dimensions respectively. There are no hydrogen bonds in the structure of compound (I) other than those within the selected asymmetric unit (Table 1 ▸, Fig. 1 ▸). However, the hydrogen-bonded ionic components are linked into a chain by an aromatic π–π stacking inter­action. The C5*A*,C6–C9,C9*A* ring in the cation at (*x*, *y*, *z*) makes a dihedral angle of only 1.34 (12)° with the C21–C26 ring in the anion at (*x*, 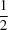 − *y*, 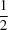 + *z*). The distance between the centroids of these two rings is 3.4583 (14) Å and the shortest perpendicular distance between the centroid of one ring and the plane of the other is 3.2761 (1) Å, corresponding to a ring-centroid offset of *ca* 1.11 Å. This stacking inter­action links the hydrogen-bonded ionic components into a chain running parallel to the [001] direction (Fig. 4 ▸). Two chains of this type, related to one another by inversion, pass through each unit cell, but there are no direction-specific inter­actions between adjacent chains. The DMSO mol­ecules are pendent from the chains but otherwise play no part in the supra­molecular assembly, so that their role may be largely that of filling otherwise empty cavities within the structure formed by the ionic components.

**Figure 5:**
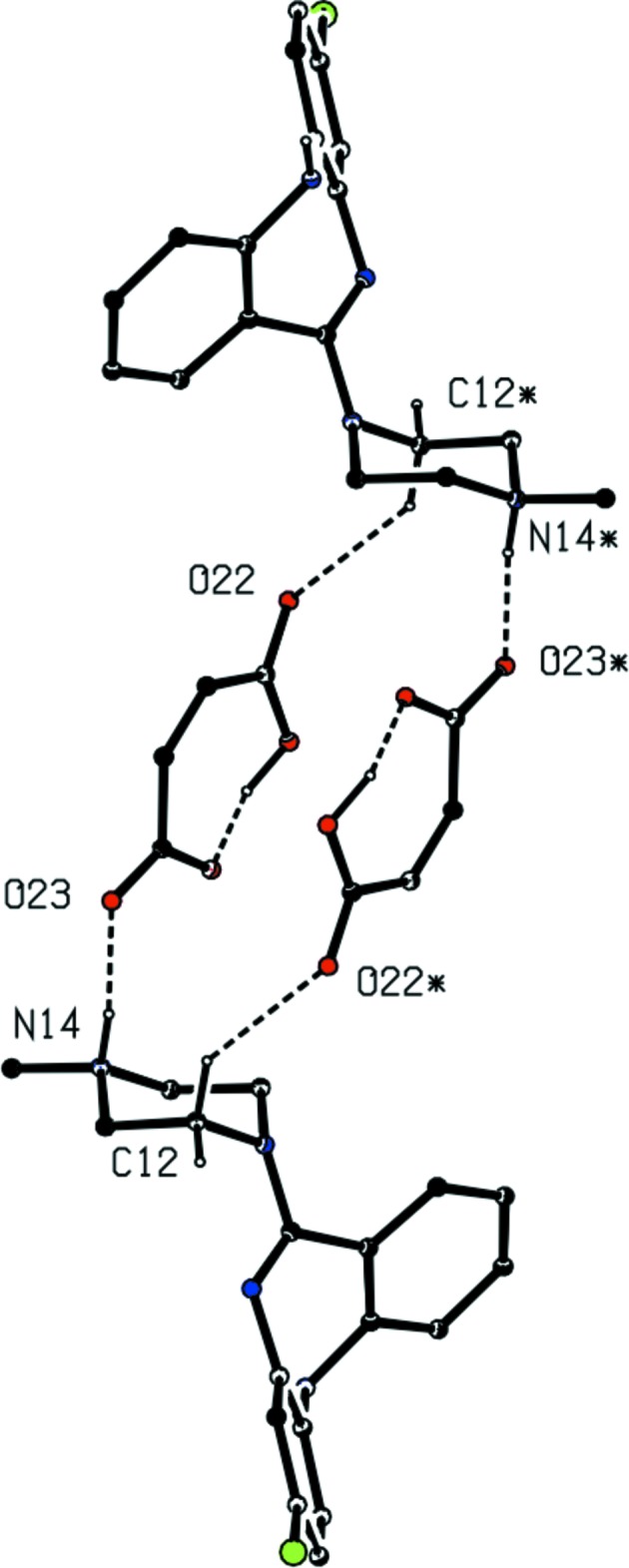
Part of the crystal structure of compound (II) showing the formation of a centrosymmetric four-ion aggregate. For the sake of clarity, the unit-cell outline and the H atoms bonded to C atoms not involved in the motif shown have been omitted. Atoms marked with an asterisk (*) are at the symmetry position (2 − *x*, 1 − *y*, 1 − *z*).

**Figure 6:**
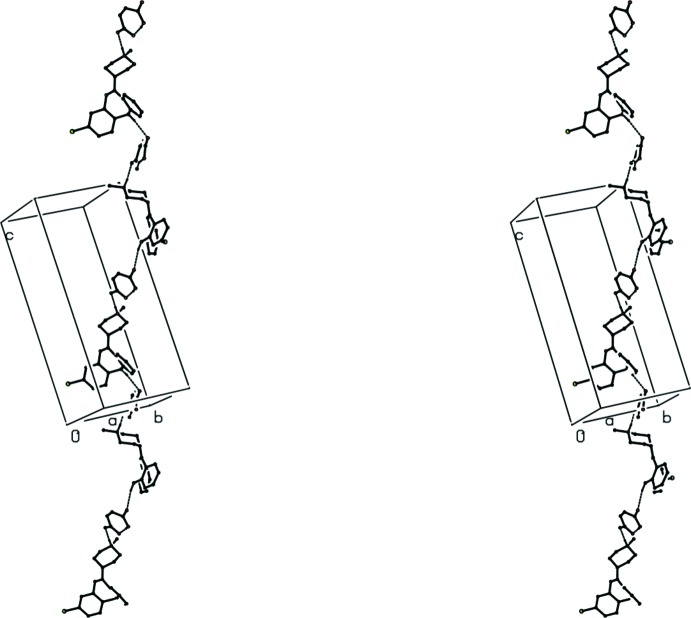
A stereoview of part of the crystal structure of compound (II) showing the formation of hydrogen-bonded 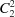
(16) chain parallel to [201]. For the sake of clarity, H atoms bonded to C atoms and the partial-occupancy water mol­ecules have been omitted.

**Figure 7:**
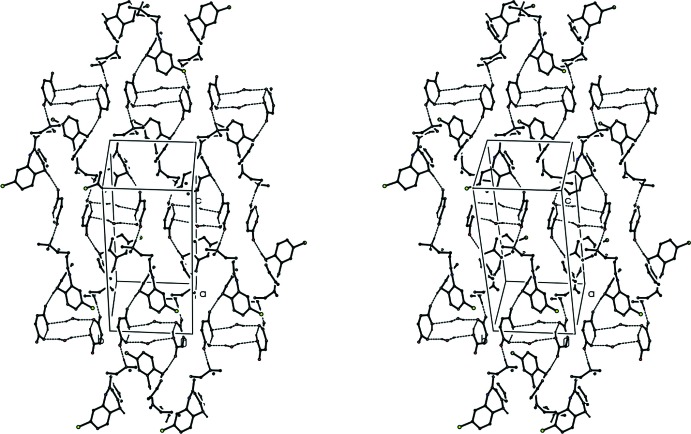
A stereoview of part of the crystal structure of compound (II) showing the formation of a hydrogen-bonded sheet lying parallel to (10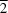). For the sake of clarity, H atoms bonded to C atoms not involved in the motifs shown have been omitted.

In compound (II) a combination of O—H⋯O, N—H⋯O and C—H⋯O hydrogen bonds (Table 1 ▸) links the independent components into complex sheets, but the sheet formation can readily be analysed in terms of a small number of fairly simple sub-structures (Ferguson *et al.*, 1998*a* ▸,*b* ▸; Gregson *et al.*, 2000 ▸). Ion pairs (Fig. 2 ▸) which are related by inversion are linked by C—H⋯O hydrogen bonds, forming a cyclic centrosymmetric aggregate characterized by an 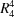
(22) (Bernstein *et al.*, 1995 ▸) motif, with the reference aggregate centred at (1, 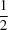, 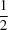) (Fig. 5 ▸). In a second sub-structure, ion pairs which are related by a glide plane are linked by N—H⋯O hydrogen bonds to form a 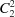
(16) chain running parallel to the [201] direction (Fig. 6 ▸). This chain motif directly links the reference four-ion aggregate (Fig. 5 ▸) centred at (1, 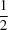, 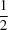) to the four symmetry-related aggregates centred at (0, 0, 0), (0, 1, 0), (2, 0, 1) and (2, 1, 1), so forming a sheet lying parallel to (1 0 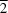) (Fig. 7 ▸). Embedded within this sheet is a further cyclic motif, which is formally centrosymmetric, containing two anions and two water mol­ecules. However, since the occupancy of the water sites is only 0.210 (7), if either of the two water sites in this motif is occupied there is a high probability that the other such site will be unoccupied: indeed, in the majority of cases, neither site will be occupied.

**Figure 8:**
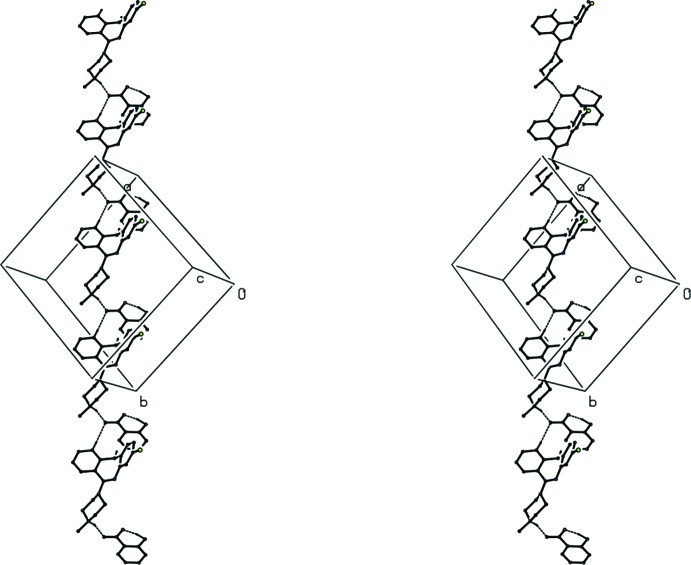
A stereoview of part of the crystal structure of compound (III) showing the formation of a hydrogen-bonded 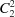
(11) chain running parallel to [1
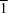
0]. For the sake of clarity, H atoms not involved in the motif shown have been omitted.

**Figure 9:**
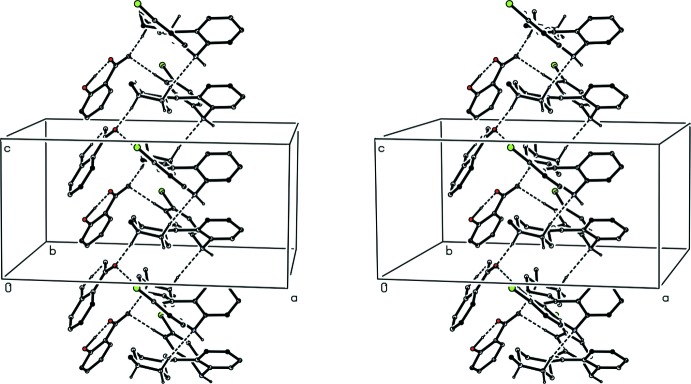
A stereoview of part of the crystal structure of compound (III) showing the formation of a hydrogen-bonded *C*(7) 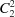
(11)[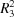
(19)] chain of rings running parallel to [001]. For the sake of clarity, H atoms bonded to the C atoms not involved in the motif shown have been omitted.

**Figure 10:**
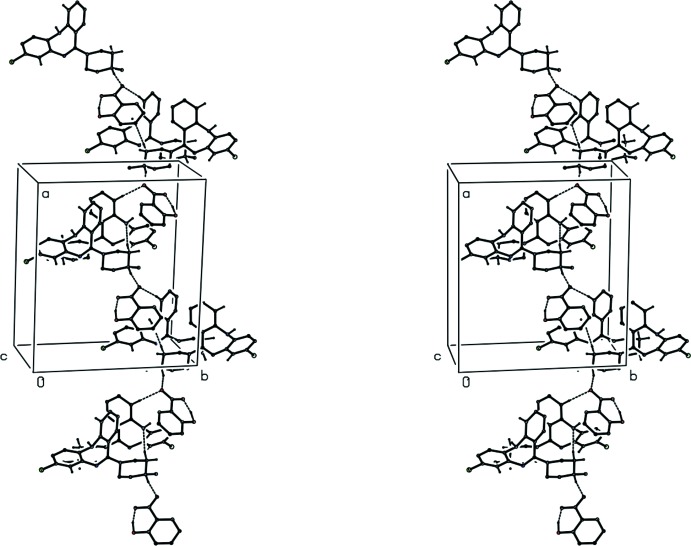
A stereoview of part of the crystal structure of compound (III) showing the formation of a hydrogen-bonded chain running parallel to [101]. For the sake of clarity, H atoms bonded to the C atoms not involved in the motif shown have been omitted.

The independent components of compound (III) are linked into a three-dimensional framework structure by a combination of N—H⋯O, O—H⋯O and C—H⋯N hydrogen bonds (Table 1 ▸), and again the formation of the framework is most readily analysed in terms of three one-dimensional substructures. In the simplest of these sub-structures, the C—H⋯O hydrogen bond involving atom C4 links ion pairs related by translation into a 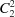
(11) chain running parallel to the [1
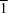
0] direction (Fig. 8 ▸). The second sub-structure involves both C—H⋯N and C—H⋯O hydrogen bonds: cations related by the *c*-glide plane at *y* = 0.5 are linked by C—H⋯N hydrogen bonds into *C*(7) chains running parallel to the [001] direction, and similarly related ion pairs are linked by the C—H⋯O hydrogen bond involving atom C9 to form a 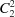
(11) chain also running parallel to [001], such that the combined effect of these two hydrogen bonds generates a *C*(7) 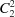
(11)[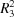
(19)] chain of rings running parallel to [001] (Fig. 9 ▸). Finally, the alternating action of the hydrogen bonds involving, atom C4 on the one hand, and atoms C9 and C15 on the other (Table 1 ▸) generates a complex chain running parallel to the [101] direction (Fig. 10 ▸).

## Database survey   

It is of inter­est briefly to compare the structures reported here for the salts (I)–(III) with those of some closely related analogues, in particular clozapine itself, compound (IV) (see Scheme 2) and the di­hydro­bromide salt (V). 
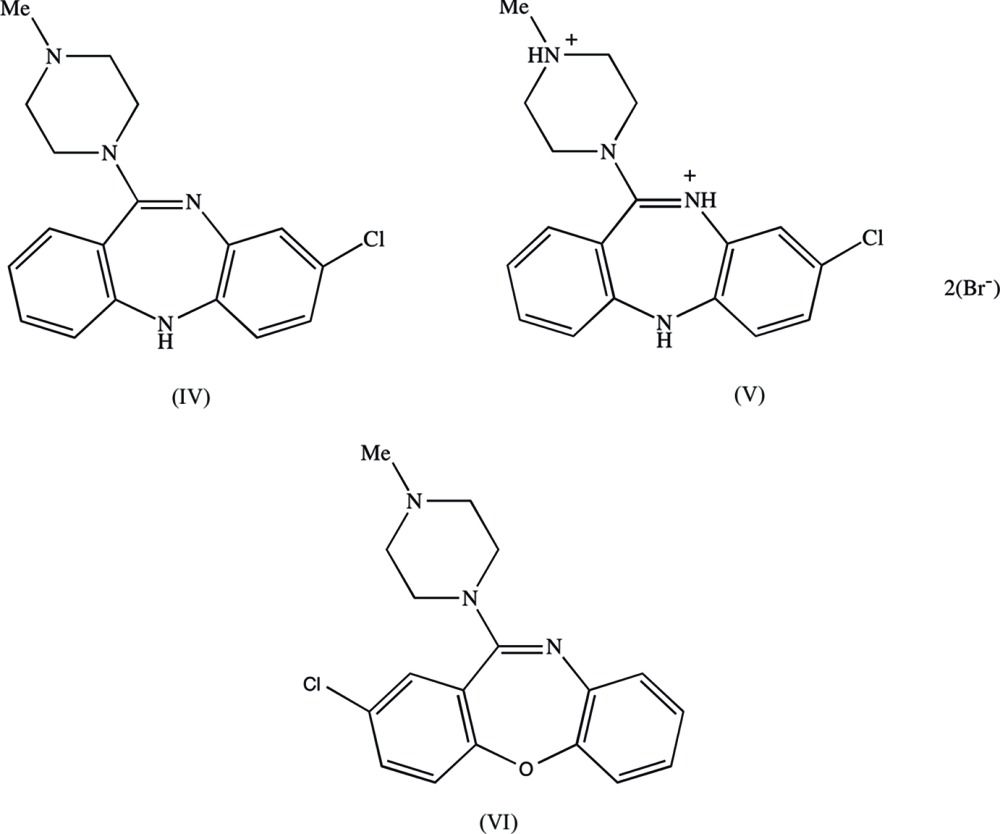


In the free base clozapine, which crystallizes in the space group *P*2_1_2_1_2_1_ (Petcher & Weber, 1976 ▸), the geometry at the piperazinyl N atom corresponding to atom N11 in compounds (I)–(III) is very nearly planar, with a sum of inter­bond angles of 357 (2)°, and there are no direction-specific inter­actions between the mol­ecules: in particular, the N—H bond does not participate in any kind of hydrogen bond. In the salt (V), which crystallizes in space group *P*2_1_/*c* (Fillers & Hawkinson, 1982 ▸), the protonation sites are the N atoms corresponding to atoms N10 and N14 in compounds (I)–(III), so that the doubly bonded N atom of the diazepine ring is protonated in preference to the second N atom of the piperazine ring where, as in (IV), the geometry in nearly planar, with a sum of inter­bond angles of 357 (2)°: the individual ionic components in (V) are linked by charge-assisted N—H⋯Br hydrogen bonds, such that cations related by a 2_1_ screw axis are bridged by one of the two independent anions to form a 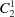
(7) chain, from which the anions of the second type are pendent. Loxapine, compound (VI), is similar to clozapine but differs from it in two respects: the nature of the hetero-atoms in the seven-membered ring, and the location of the Cl substituent. Here again there are no direction-specific inter­actions between the mol­ecules (Petcher & Weber, 1976 ▸). The overall mol­ecular shapes of the mol­ecules of compounds (IV) and (VI) are extremely similar, and it was suggested (Petcher & Weber, 1976 ▸) that the structures observed in the solid state represented a preferred form which persists in aqueous solutions and at the site of neuroleptic receptors. However, in the presence of charge-assisted hydrogen bonds in compounds (I)–(III), reported here, which are probably slightly stronger that those between the mol­ecules of (IV) and (VI) and adjacent water mol­ecules in solution, the mol­ecular configurations show some significant differences, as noted above, so that no preferred configuration is apparent from the structures of (I)–(III).

## Synthesis and crystallization   

Clozapine was a gift from R L Fine Chem, Bengaluru, Karnataka, India. Equimolar qu­anti­ties of clozapine and the appropriate acid (1.53 mmol of each component) were dissolved in methanol at 333 K. The solutions were permitted to cool to ambient temperature, and the resulting crystals were then collected by filtration, and dried over phospho­rus(V) oxide. Crystals suitable for single-crystal X-ray diffraction were obtained by slow evaporation, at ambient temperature and in the presence of air, of solutions in dimethyl sulfoxide, for compounds (I) and (II), and a mixture (1:1 *v*/*v*) of aceto­nitrile and methanol for compound (III).

## Refinement   

Crystal data, data collection and structure refinement details are summarized in Table 3 ▸. The H atoms bonded to C or N atoms in the ionic components of compounds (I)–(III) were all located in difference maps. The H atoms bonded to C atoms were then treated as riding atoms in geometrically idealized positions with C—H distances of 0.95 Å (alkenyl and aromatic), 0.98 Å (CH_3_) or 0.99 Å (CH_2_) and with *U*_iso_(H) = *kU*_eq_(C), where *k* = 1.5 for the methyl groups, which were permitted to rotate but not to tilt, and 1.2 for all other H atoms bonded to C atoms. For the H atoms bonded to N or O atoms, the atomic coordinates were refined with *U*_iso_(H) = 1.2*U*_eq_(N) or 1.5*U*_eq_(O), giving the N—H and O—H distances shown in Table 1 ▸.

In compound (I), the dimethyl sulfoxide component is disordered over two sets of atomic sites having unequal occupancy (*cf.* Fig. 1 ▸). For the minor disorder component, the bonded distances and the one-angle non-bonded distances were all restrained to be the same as the corresponding distances in the major component subject to uncertainties of 0.005 Å and 0.01 Å respectively. The anisotropic displacement parameters for those pairs of partial-occupancy C and O atoms occupying essentially the same physical space were constrained to be identical, and the H atoms of the dimethyl sulfoxide components were included as riding atoms with C—H distances 0.95 Å and *U*_iso_(H) = 1.5*U*_eq_(C). Subject to these conditions, independent refinement of the site occupancies for the two disorder components gave values of 0.613 (3) and 0.359 (3): thereafter the occupancies were constrained to sum to unity, giving final values of 0.627 (2) and 0.373 (2). At this stage of the refinements there were no significant features in the difference maps for compounds (I) and (III), but for (II) there was a single significant peak, 1.51 e Å^−3^, which was within plausible hydrogen-bonding distance of two O atoms. Examination of the structures of compounds (I) and (III) using *PLATON* (Spek, 2009 ▸) showed that there were no solvent-accessible voids in these structures. However, in compound (II), there was a total void volume of *ca* 88 Å^3^ per unit cell, and examination of the structure of (II) using the SQUEEZE tool (Spek, 2015 ▸) within *PLATON* disclosed the presence of an addition 8.8 electrons per unit cell, equivalent to 0.22 mol­ecules of water per ion pair. Accordingly, the large residual was modeled as the O atom, denoted O31, of a partial occupancy water mol­ecule, which was refined isotropically: it was not possible to locate the H atoms associated with atom O31 in difference maps, but they were included in calculated positions with O—H 0.90 Å and *U*_iso_(H) = 1.5*U*_iso_(O). Subject to these conditions, the occupancy of the water mol­ecule refined to a value of 0.210 (7), very close to that indicated by SQUEEZE. It should be emphasized here that the application of the SQUEEZE procedure referred to above was intended only to estimate the number of electrons not yet accounted for at that stage of the refinement, and that the refinements at every stage were undertaken with the original data, independent of SQUEEZE. For compound (III), the correct orientation of the structure with respect to the polar axis directions was established by means of the Flack *x* parameter (Flack, 1983 ▸), *x* = −0.022 (17), calculated (Parsons *et al.*, 2013 ▸) using 1674 quotients of the type [(*I* ^+^)−(*I* ^−^)]/[(*I* ^+^)+(*I* ^−^)].

## Supplementary Material

Crystal structure: contains datablock(s) global, I, II, III. DOI: 10.1107/S205698901500554X/hb7383sup1.cif

Structure factors: contains datablock(s) I. DOI: 10.1107/S205698901500554X/hb7383Isup2.hkl

Structure factors: contains datablock(s) II. DOI: 10.1107/S205698901500554X/hb7383IIsup3.hkl

Structure factors: contains datablock(s) III. DOI: 10.1107/S205698901500554X/hb7383IIIsup4.hkl

CCDC references: 1054724, 1054723, 1054722

Additional supporting information:  crystallographic information; 3D view; checkCIF report

## Figures and Tables

**Table 1:** Hydrogen bonds and short intra­molecular contacts (Å, °) for compounds (I)–(III) *Cg*1 and *Cg*2 represent the centroids of rings C5*A*/C6–C9/C9*A* and C1–C4/C4*A*/C11*A*, respectively

Compound	*D*—H⋯*A*		*D*—H	H⋯*A*	*D*⋯*A*	*D*—H⋯*A*
(I)	N5—H5⋯O31		0.83 (3)	2.10 (3)	2.921 (4)	170 (3)
	N14—H14⋯O22		1.00 (3)	1.58 (3)	2.575 (3)	176 (2)
	C4—H4⋯O31		0.95	2.58	3.342 (4)	137
	C4—H4⋯O41		0.95	2.38	3.208 (7)	146
	C6—H6⋯O31		0.95	2.54	3.313 (5)	139
(II)	N5—H5⋯O22^i^		0.86 (3)	2.24 (3)	3.084 (2)	170 (3)
	N14—H14⋯O23		0.87 (3)	1.83 (2)	2.688 (2)	173 (2)
	O21—H21⋯O24		1.00 (5)	1.47 (5)	2.420 (3)	157 (5)
	O31—H31*A*⋯O24		0.90	2.05	2.913 (11)	160
	O31—H31*B*⋯O21^ii^		0.90	2.37	3.232 (11)	161
	C12—H12*A*⋯O22^iii^		0.99	2.48	3.308 (2)	141
	C15—H15*A*⋯*Cg*1^iv^		0.99	2.95	3.642 (2)	128
	C15—H15*B*⋯*Cg*2^iv^		0.99	2.95	3.759 (2)	139
(III)	N14—H14⋯O22		0.89 (6)	1.75 (6)	2.612 (4)	164 (5)
	O23—H23⋯O21		1.01 (11)	1.52 (10)	2.507 (5)	161 (9)
	C4—H4⋯O22^v^		0.95	2.23	3.261 (4)	166
	C9—H9⋯O22^v^		0.95	2.25	3.202 (4)	176
	C12—H12*A*⋯O21		0.99	2.44	3.306 (5)	146
	C12—H12*B*⋯N5^vi^		0.99	2.56	3.539 (4)	170
	C24—H24⋯*Cg*1^vii^		0.95	2.83	3.637 (5)	144

Symmetry codes: (i) −1 + *x*, 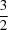 − *y*, −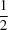 + *z*; (ii) −*x*, 2 − *y*, 1 − *z*; (iii) 2 − *x*, 1 − *y*, 1 − *z*; (iv) 1 − *x*, 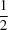 + *y*, 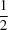 − *z*; (v) 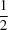 + *x*, −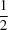 + *y*, *z*; (vi) *x*, 1 − *y*, 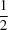 + *z*; (vii) −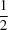 + *x*, 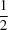 + *y*, −1 + *z*.

**Table 2:** Selected geometric parameters (°) for compounds (I)–(III) ‘Dihedral’ denotes the dihedral angles between the mean planes of rings C1–C4/C4*A*/C11*A* and C5*A*/C6–C9/C9*A*.

Parameter		(I)	(II)	(III)
C11—N11—C12		120.80 (19)	118.45 (15)	118.3 (3)
C11—N11—C16		126.01 (19)	122.04 (15)	121.7 (3)
C12—N11—C16		112.91 (18)	111.09 (14)	111.0 (3)
C4*A*—N5—C5*A*—C6		−117.8 (2)	−116.17 (19)	−117.1 (3)
C5*A*—N5—C4*A*—C4		115.1 (3)	120.07 (19))	115.3 (3)
C1—C11*A*—C11—N10		−129.2 (3)	−137.5 (2)	−139.6 (3)
C9—C9*A*—N10—C11		146.3 (2)	141.11 (19)	141.9 (3)
C9*A*—N10—C11—C11*A*		−7.9 (4)	0.9 (3)	1.0 (5)
N10—C11—N11—C12		6.3 (3)	2.7 (3)	5.5 (4)
C11—N11—C12—C13		−120.8 (2)	−90.0 (2)	151.8 (3)
Dihedral		62.21 (11)	60.97 (9)	59.07 (16)

**Table 3:** Experimental details

	(I)	(II)	(III)
Crystal data			
Chemical formula	C_18_H_20_ClN_4_^+^·C_7_H_3_N_2_O_6_^−^·C_2_H_6_OS	C_18_H_20_ClN_4_^+^·C_4_H_3_O_4_^−^·0.21H_2_O	C_18_H_20_ClN_4_^+^·C_7_H_5_O_3_^−^
*M*_r_	617.07	446.68	464.94
Crystal system, space group	Monoclinic, *P*2_1_/*c*	Monoclinic, *P*2_1_/*c*	Monoclinic, *C* *c*
Temperature (K)	173	173	173
*a*, *b*, *c* (Å)	15.3593 (2), 15.7685 (2), 11.8679 (2)	9.7166 (3), 9.9699 (2), 23.1059 (6)	17.4296 (5), 15.3728 (5), 8.6359 (3)
β (°)	91.8097 (14)	96.800 (3)	90.325 (3)
*V* (Å^3^)	2872.89 (7)	2222.60 (10)	2313.88 (13)
*Z*	4	4	4
Radiation type	Cu *K*α	Cu *K*α	Cu *K*α
μ (mm^−1^)	2.34	1.84	1.75
Crystal size (mm)	0.26 × 0.22 × 0.18	0.46 × 0.32 × 0.22	0.42 × 0.36 × 0.20

Data collection			
Diffractometer	Agilent Eos Gemini	Agilent Eos Gemini	Agilent Eos Gemini
Absorption correction	Multi-scan (*CrysAlis RED*; Agilent, 2012 ▸)	Multi-scan (*CrysAlis RED*; Agilent, 2012 ▸)	Multi-scan (*CrysAlis RED*; Agilent, 2012 ▸)
*T*_min_, *T*_max_	0.424, 0.656	0.440, 0.668	0.399, 0.705
No. of measured, independent and observed [*I* > 2σ(*I*)] reflections	19784, 5527, 4623	8634, 4228, 3552	7244, 4069, 3962
*R*_int_	0.032	0.026	0.048
(sin θ/λ)_max_ (Å^−1^)	0.614	0.614	0.619

Refinement			
*R*[*F* ^2^ > 2σ(*F* ^2^)], *wR*(*F* ^2^), *S*	0.056, 0.152, 1.04	0.048, 0.133, 1.03	0.056, 0.141, 1.08
No. of reflections	5527	4228	4069
No. of parameters	409	295	308
No. of restraints	6	0	2
H-atom treatment	H atoms treated by a mixture of independent and constrained refinement	H atoms treated by a mixture of independent and constrained refinement	H atoms treated by a mixture of independent and constrained refinement
Δρ_max_, Δρ_min_ (e Å^−3^)	0.89, −0.97	0.46, −0.30	0.53, −0.36
Absolute structure	–	–	Flack *x* determined using 1674 quotients [(*I* ^+^)−(*I* ^−^)]/[(*I* ^+^)+(*I* ^−^)] (Parsons *et al.*, 2013 ▸)
Absolute structure parameter	–	–	−0.022 (17)

Computer programs: *CrysAlis PRO* and *CrysAlis RED* (Agilent, 2012 ▸), *SHELXS97* (Sheldrick, 2008 ▸), *SHELXL2014* (Sheldrick, 2015 ▸) and *PLATON* (Spek, 2009 ▸).

## supplementary crystallographic information

### (I) Clozapinium 3,5-dinitrobenzoate dimethyl sulfoxide monosolvate Crystal data

**Table:**

C_18_H_20_ClN_4_^+^·C_7_H_3_N_2_O_6_^−^·C_2_H_6_OS	*F*(000) = 1288
*M**_r_* = 617.07	*D*_x_ = 1.427 Mg m^−^^3^
Monoclinic, *P*2_1_/*c*	Cu *K*α radiation, λ = 1.54184 Å
*a* = 15.3593 (2) Å	Cell parameters from 5527 reflections
*b* = 15.7685 (2) Å	θ = 4.0–71.3°
*c* = 11.8679 (2) Å	µ = 2.34 mm^−^^1^
β = 91.8097 (14)°	*T* = 173 K
*V* = 2872.89 (7) Å^3^	Block, colourless
*Z* = 4	0.26 × 0.22 × 0.18 mm

### (I) Clozapinium 3,5-dinitrobenzoate dimethyl sulfoxide monosolvate Data collection

**Table:**

Agilent Eos Gemini diffractometer	4623 reflections with *I* > 2σ(*I*)
Radiation source: Enhance (Cu) X-ray Source	*R*_int_ = 0.032
ω scans	θ_max_ = 71.3°, θ_min_ = 4.0°
Absorption correction: multi-scan (CrysAlis RED; Agilent, 2012)	*h* = −17→18
*T*_min_ = 0.424, *T*_max_ = 0.656	*k* = −19→15
19784 measured reflections	*l* = −14→14
5527 independent reflections	

### (I) Clozapinium 3,5-dinitrobenzoate dimethyl sulfoxide monosolvate Refinement

**Table:**

Refinement on *F*^2^	6 restraints
Least-squares matrix: full	Hydrogen site location: difference Fourier map
*R*[*F*^2^ > 2σ(*F*^2^)] = 0.056	H atoms treated by a mixture of independent and constrained refinement
*wR*(*F*^2^) = 0.152	*w* = 1/[σ^2^(*F*_o_^2^) + (0.0639*P*)^2^ + 2.7986*P*] where *P* = (*F*_o_^2^ + 2*F*_c_^2^)/3
*S* = 1.04	(Δ/σ)_max_ < 0.001
5527 reflections	Δρ_max_ = 0.89 e Å^−^^3^
409 parameters	Δρ_min_ = −0.97 e Å^−^^3^

### (I) Clozapinium 3,5-dinitrobenzoate dimethyl sulfoxide monosolvate Special details

**Table:**

Geometry. All e.s.d.'s (except the e.s.d. in the dihedral angle between two l.s. planes) are estimated using the full covariance matrix. The cell e.s.d.'s are taken into account individually in the estimation of e.s.d.'s in distances, angles and torsion angles; correlations between e.s.d.'s in cell parameters are only used when they are defined by crystal symmetry. An approximate (isotropic) treatment of cell e.s.d.'s is used for estimating e.s.d.'s involving l.s. planes.

### (I) Clozapinium 3,5-dinitrobenzoate dimethyl sulfoxide monosolvate Fractional atomic coordinates and isotropic or equivalent isotropic displacement parameters (Å^2^)

**Table:**

	*x*	*y*	*z*	*U*_iso_*/*U*_eq_	Occ. (<1)
C1	0.35763 (15)	0.19065 (15)	0.64680 (19)	0.0349 (5)	
H1	0.3526	0.1546	0.5828	0.042*	
C2	0.40839 (15)	0.16561 (16)	0.7394 (2)	0.0396 (5)	
H2	0.4388	0.1132	0.7385	0.048*	
C3	0.41440 (16)	0.21776 (18)	0.8335 (2)	0.0428 (6)	
H3	0.4452	0.1990	0.8996	0.051*	
C4	0.37586 (16)	0.29647 (17)	0.8315 (2)	0.0406 (5)	
H4	0.3830	0.3332	0.8945	0.049*	
C4A	0.32657 (14)	0.32265 (15)	0.7379 (2)	0.0344 (5)	
N5	0.28834 (14)	0.40442 (13)	0.73413 (19)	0.0398 (5)	
H5	0.296 (2)	0.4270 (19)	0.797 (3)	0.048*	
C5A	0.32343 (14)	0.45611 (15)	0.6491 (2)	0.0374 (5)	
C6	0.36736 (17)	0.53078 (17)	0.6785 (3)	0.0479 (6)	
H6	0.3744	0.5458	0.7557	0.057*	
C7	0.40067 (17)	0.58291 (18)	0.5976 (3)	0.0534 (7)	
H7	0.4290	0.6345	0.6180	0.064*	
C8	0.39217 (16)	0.55887 (17)	0.4864 (3)	0.0491 (7)	
Cl8	0.43801 (6)	0.62363 (5)	0.38475 (8)	0.0715 (3)	
C9	0.34849 (16)	0.48559 (16)	0.4543 (2)	0.0422 (6)	
H9	0.3430	0.4709	0.3767	0.051*	
C9A	0.31242 (14)	0.43313 (14)	0.5354 (2)	0.0355 (5)	
N10	0.25884 (12)	0.36645 (12)	0.49775 (17)	0.0361 (4)	
C11	0.25516 (14)	0.29455 (14)	0.54945 (19)	0.0333 (5)	
C11A	0.31376 (14)	0.26801 (14)	0.64620 (19)	0.0328 (5)	
N11	0.19690 (14)	0.23520 (13)	0.50890 (17)	0.0393 (5)	
C12	0.14714 (16)	0.24954 (16)	0.4036 (2)	0.0399 (5)	
H12A	0.1621	0.2052	0.3484	0.048*	
H12B	0.1634	0.3052	0.3720	0.048*	
C13	0.05023 (16)	0.24786 (15)	0.4213 (2)	0.0396 (5)	
H13A	0.0340	0.2952	0.4712	0.047*	
H13B	0.0184	0.2551	0.3480	0.047*	
N14	0.02552 (12)	0.16556 (12)	0.47312 (16)	0.0344 (4)	
H14	0.0420 (18)	0.1196 (18)	0.420 (2)	0.041*	
C15	0.07363 (16)	0.15561 (16)	0.5833 (2)	0.0387 (5)	
H15A	0.0576	0.1010	0.6181	0.046*	
H15B	0.0570	0.2018	0.6348	0.046*	
C16	0.17085 (16)	0.15805 (15)	0.5671 (2)	0.0375 (5)	
H16A	0.2016	0.1551	0.6415	0.045*	
H16B	0.1882	0.1080	0.5227	0.045*	
C17	−0.06991 (17)	0.1565 (2)	0.4843 (3)	0.0531 (7)	
H17A	−0.0826	0.1017	0.5190	0.080*	
H17B	−0.0987	0.1595	0.4095	0.080*	
H17C	−0.0915	0.2024	0.5316	0.080*	
C21	0.10211 (14)	−0.00223 (14)	0.1606 (2)	0.0328 (5)	
C22	0.14232 (15)	−0.07486 (15)	0.2025 (2)	0.0392 (5)	
H22	0.1445	−0.0862	0.2811	0.047*	
C23	0.17913 (16)	−0.13049 (15)	0.1277 (3)	0.0457 (6)	
C24	0.17770 (16)	−0.11803 (18)	0.0129 (3)	0.0483 (7)	
H24	0.2032	−0.1573	−0.0370	0.058*	
C25	0.13710 (15)	−0.04533 (18)	−0.0255 (2)	0.0431 (6)	
C26	0.09975 (14)	0.01300 (15)	0.0456 (2)	0.0362 (5)	
H26	0.0729	0.0628	0.0158	0.043*	
C27	0.06407 (15)	0.06224 (15)	0.2402 (2)	0.0359 (5)	
O21	0.03166 (12)	0.12736 (11)	0.19957 (16)	0.0466 (4)	
O22	0.07009 (12)	0.04261 (11)	0.34434 (15)	0.0442 (4)	
N23	0.22603 (19)	−0.20507 (16)	0.1753 (3)	0.0687 (8)	
O23	0.2293 (3)	−0.21397 (19)	0.2776 (3)	0.1090 (11)	
O24	0.26036 (17)	−0.25228 (15)	0.1091 (3)	0.0895 (9)	
N25	0.13695 (16)	−0.0268 (2)	−0.1471 (2)	0.0619 (7)	
O25	0.17166 (18)	−0.0786 (2)	−0.2086 (2)	0.1021 (11)	
O26	0.10244 (16)	0.03914 (18)	−0.18044 (18)	0.0696 (7)	
S31	0.31808 (7)	0.56967 (6)	1.00103 (8)	0.0448 (3)	0.627 (2)
O31	0.3389 (3)	0.4857 (2)	0.9475 (3)	0.0655 (11)	0.627 (2)
C31	0.4020 (7)	0.6409 (4)	0.9689 (7)	0.083 (3)	0.627 (2)
H31A	0.3963	0.6574	0.8894	0.125*	0.627 (2)
H31B	0.4586	0.6135	0.9830	0.125*	0.627 (2)
H31C	0.3979	0.6914	1.0165	0.125*	0.627 (2)
C32	0.3429 (6)	0.5547 (4)	1.1451 (4)	0.081 (2)	0.627 (2)
H32A	0.4050	0.5416	1.1557	0.122*	0.627 (2)
H32B	0.3082	0.5076	1.1735	0.122*	0.627 (2)
H32C	0.3293	0.6066	1.1865	0.122*	0.627 (2)
S41	0.39889 (18)	0.53363 (13)	1.05795 (17)	0.0678 (8)	0.373 (2)
O41	0.3879 (5)	0.4760 (4)	0.9582 (6)	0.0655 (11)	0.373 (2)
C41	0.4247 (12)	0.6356 (6)	1.0098 (12)	0.083 (3)	0.373 (2)
H41A	0.3746	0.6590	0.9670	0.125*	0.373 (2)
H41B	0.4749	0.6323	0.9610	0.125*	0.373 (2)
H41C	0.4388	0.6723	1.0744	0.125*	0.373 (2)
C42	0.2944 (5)	0.5526 (6)	1.1067 (8)	0.081 (2)	0.373 (2)
H42A	0.2725	0.5012	1.1426	0.122*	0.373 (2)
H42B	0.2554	0.5683	1.0432	0.122*	0.373 (2)
H42C	0.2967	0.5989	1.1618	0.122*	0.373 (2)

### (I) Clozapinium 3,5-dinitrobenzoate dimethyl sulfoxide monosolvate Atomic displacement parameters (Å^2^)

**Table:**

	*U*^11^	*U*^22^	*U*^33^	*U*^12^	*U*^13^	*U*^23^
C1	0.0350 (11)	0.0347 (11)	0.0348 (11)	0.0028 (9)	−0.0013 (9)	−0.0032 (9)
C2	0.0350 (12)	0.0400 (13)	0.0437 (13)	0.0097 (10)	−0.0005 (10)	0.0000 (10)
C3	0.0363 (12)	0.0554 (15)	0.0363 (12)	0.0065 (11)	−0.0053 (10)	0.0002 (11)
C4	0.0372 (12)	0.0489 (14)	0.0357 (12)	0.0012 (11)	−0.0011 (10)	−0.0089 (10)
C4A	0.0283 (11)	0.0352 (12)	0.0398 (12)	0.0012 (9)	0.0030 (9)	−0.0039 (9)
N5	0.0391 (11)	0.0353 (11)	0.0452 (12)	0.0046 (9)	0.0042 (9)	−0.0088 (9)
C5A	0.0257 (10)	0.0322 (11)	0.0543 (14)	0.0039 (9)	0.0006 (10)	−0.0038 (10)
C6	0.0348 (13)	0.0416 (14)	0.0670 (17)	−0.0027 (11)	−0.0037 (12)	−0.0089 (12)
C7	0.0365 (13)	0.0398 (14)	0.084 (2)	−0.0080 (11)	−0.0040 (13)	−0.0051 (14)
C8	0.0301 (12)	0.0383 (13)	0.079 (2)	−0.0014 (10)	0.0071 (12)	0.0105 (13)
Cl8	0.0632 (5)	0.0533 (4)	0.0991 (6)	−0.0144 (4)	0.0176 (4)	0.0167 (4)
C9	0.0332 (12)	0.0361 (12)	0.0573 (15)	0.0052 (10)	0.0037 (11)	0.0031 (11)
C9A	0.0237 (10)	0.0291 (11)	0.0534 (14)	0.0042 (9)	−0.0015 (9)	0.0004 (10)
N10	0.0305 (9)	0.0315 (10)	0.0459 (11)	0.0015 (8)	−0.0031 (8)	0.0014 (8)
C11	0.0293 (11)	0.0331 (11)	0.0373 (12)	0.0029 (9)	−0.0015 (9)	−0.0012 (9)
C11A	0.0288 (10)	0.0328 (11)	0.0365 (11)	0.0005 (9)	−0.0016 (9)	−0.0009 (9)
N11	0.0423 (11)	0.0353 (10)	0.0394 (10)	−0.0076 (9)	−0.0112 (9)	0.0057 (8)
C12	0.0437 (13)	0.0390 (13)	0.0362 (12)	−0.0066 (10)	−0.0098 (10)	0.0055 (10)
C13	0.0435 (13)	0.0321 (12)	0.0426 (13)	0.0041 (10)	−0.0073 (10)	0.0016 (10)
N14	0.0316 (10)	0.0342 (10)	0.0373 (10)	0.0035 (8)	−0.0008 (8)	−0.0004 (8)
C15	0.0434 (13)	0.0372 (12)	0.0354 (12)	0.0004 (10)	−0.0015 (10)	0.0017 (10)
C16	0.0402 (12)	0.0327 (12)	0.0389 (12)	−0.0025 (10)	−0.0094 (10)	0.0053 (9)
C17	0.0336 (13)	0.0621 (18)	0.0636 (18)	0.0058 (12)	0.0029 (12)	0.0058 (14)
C21	0.0254 (10)	0.0292 (11)	0.0436 (12)	−0.0045 (9)	−0.0015 (9)	−0.0033 (9)
C22	0.0352 (12)	0.0313 (11)	0.0510 (14)	−0.0026 (10)	−0.0029 (10)	−0.0001 (10)
C23	0.0332 (12)	0.0296 (12)	0.0742 (19)	−0.0001 (10)	−0.0026 (12)	−0.0076 (12)
C24	0.0305 (12)	0.0480 (15)	0.0665 (18)	−0.0061 (11)	0.0048 (11)	−0.0243 (13)
C25	0.0290 (11)	0.0542 (15)	0.0459 (14)	−0.0113 (11)	0.0010 (10)	−0.0122 (11)
C26	0.0262 (11)	0.0364 (12)	0.0458 (13)	−0.0076 (9)	−0.0025 (9)	−0.0015 (10)
C27	0.0308 (11)	0.0323 (12)	0.0445 (13)	−0.0032 (9)	−0.0008 (9)	−0.0059 (10)
O21	0.0480 (10)	0.0359 (9)	0.0554 (11)	0.0104 (8)	−0.0054 (8)	−0.0052 (8)
O22	0.0519 (10)	0.0388 (9)	0.0420 (9)	0.0027 (8)	0.0013 (8)	−0.0051 (7)
N23	0.0586 (16)	0.0345 (13)	0.113 (3)	0.0082 (12)	−0.0038 (16)	−0.0085 (15)
O23	0.142 (3)	0.0668 (18)	0.117 (3)	0.0490 (19)	−0.017 (2)	0.0159 (17)
O24	0.0714 (16)	0.0442 (12)	0.153 (3)	0.0188 (12)	0.0088 (16)	−0.0227 (15)
N25	0.0389 (12)	0.100 (2)	0.0472 (13)	−0.0174 (14)	0.0037 (10)	−0.0139 (15)
O25	0.0687 (16)	0.179 (3)	0.0587 (15)	0.0199 (19)	0.0102 (12)	−0.0409 (18)
O26	0.0705 (15)	0.0915 (18)	0.0462 (12)	−0.0312 (14)	−0.0086 (10)	0.0062 (12)
S31	0.0570 (7)	0.0362 (5)	0.0410 (5)	−0.0028 (4)	−0.0024 (4)	−0.0033 (4)
O31	0.094 (3)	0.0484 (16)	0.0543 (16)	−0.009 (2)	0.009 (2)	−0.0199 (13)
C31	0.081 (6)	0.060 (2)	0.111 (7)	−0.030 (3)	0.040 (6)	−0.037 (3)
C32	0.145 (7)	0.054 (2)	0.044 (3)	0.021 (4)	−0.006 (3)	−0.002 (2)
S41	0.109 (2)	0.0467 (11)	0.0475 (11)	0.0257 (11)	0.0001 (11)	−0.0104 (8)
O41	0.094 (3)	0.0484 (16)	0.0543 (16)	−0.009 (2)	0.009 (2)	−0.0199 (13)
C41	0.081 (6)	0.060 (2)	0.111 (7)	−0.030 (3)	0.040 (6)	−0.037 (3)
C42	0.145 (7)	0.054 (2)	0.044 (3)	0.021 (4)	−0.006 (3)	−0.002 (2)

### (I) Clozapinium 3,5-dinitrobenzoate dimethyl sulfoxide monosolvate Geometric parameters (Å, º)

**Table:**

C1—C2	1.385 (3)	C16—H16A	0.9900
C1—C11A	1.394 (3)	C16—H16B	0.9900
C1—H1	0.9500	C17—H17A	0.9800
C2—C3	1.388 (4)	C17—H17B	0.9800
C2—H2	0.9500	C17—H17C	0.9800
C3—C4	1.375 (4)	C21—C22	1.386 (3)
C3—H3	0.9500	C21—C26	1.386 (3)
C4—C4A	1.387 (3)	C21—C27	1.517 (3)
C4—H4	0.9500	C22—C23	1.381 (4)
C4A—C11A	1.397 (3)	C22—H22	0.9500
C4A—N5	1.417 (3)	C23—C24	1.376 (4)
N5—C5A	1.417 (3)	C23—N23	1.482 (4)
N5—H5	0.83 (3)	C24—C25	1.376 (4)
C5A—C6	1.396 (3)	C24—H24	0.9500
C5A—C9A	1.402 (4)	C25—C26	1.384 (4)
C6—C7	1.375 (4)	C25—N25	1.473 (4)
C6—H6	0.9500	C26—H26	0.9500
C7—C8	1.375 (4)	C27—O21	1.233 (3)
C7—H7	0.9500	C27—O22	1.274 (3)
C8—C9	1.384 (4)	N23—O24	1.215 (4)
C8—Cl8	1.746 (3)	N23—O23	1.222 (4)
C9—C9A	1.397 (4)	N25—O26	1.227 (4)
C9—H9	0.9500	N25—O25	1.228 (4)
C9A—N10	1.400 (3)	S31—O31	1.507 (3)
N10—C11	1.291 (3)	S31—C32	1.756 (5)
C11—N11	1.372 (3)	S31—C31	1.760 (5)
C11—C11A	1.496 (3)	C31—H31A	0.9800
N11—C16	1.461 (3)	C31—H31B	0.9800
N11—C12	1.461 (3)	C31—H31C	0.9800
C12—C13	1.510 (4)	C32—H32A	0.9800
C12—H12A	0.9900	C32—H32B	0.9800
C12—H12B	0.9900	C32—H32C	0.9800
C13—N14	1.491 (3)	S41—O41	1.497 (4)
C13—H13A	0.9900	S41—C42	1.749 (6)
C13—H13B	0.9900	S41—C41	1.756 (6)
N14—C17	1.483 (3)	C41—H41A	0.9800
N14—C15	1.490 (3)	C41—H41B	0.9800
N14—H14	1.00 (3)	C41—H41C	0.9800
C15—C16	1.512 (3)	C42—H42A	0.9800
C15—H15A	0.9900	C42—H42B	0.9800
C15—H15B	0.9900	C42—H42C	0.9800
			
C2—C1—C11A	120.9 (2)	N11—C16—C15	111.57 (19)
C2—C1—H1	119.5	N11—C16—H16A	109.3
C11A—C1—H1	119.5	C15—C16—H16A	109.3
C1—C2—C3	119.4 (2)	N11—C16—H16B	109.3
C1—C2—H2	120.3	C15—C16—H16B	109.3
C3—C2—H2	120.3	H16A—C16—H16B	108.0
C4—C3—C2	120.2 (2)	N14—C17—H17A	109.5
C4—C3—H3	119.9	N14—C17—H17B	109.5
C2—C3—H3	119.9	H17A—C17—H17B	109.5
C3—C4—C4A	120.5 (2)	N14—C17—H17C	109.5
C3—C4—H4	119.8	H17A—C17—H17C	109.5
C4A—C4—H4	119.8	H17B—C17—H17C	109.5
C4—C4A—C11A	120.0 (2)	C22—C21—C26	119.6 (2)
C4—C4A—N5	120.8 (2)	C22—C21—C27	120.5 (2)
C11A—C4A—N5	119.2 (2)	C26—C21—C27	119.9 (2)
C4A—N5—C5A	112.30 (19)	C23—C22—C21	118.8 (2)
C4A—N5—H5	109 (2)	C23—C22—H22	120.6
C5A—N5—H5	110 (2)	C21—C22—H22	120.6
C6—C5A—C9A	120.1 (2)	C24—C23—C22	123.5 (2)
C6—C5A—N5	119.9 (2)	C24—C23—N23	118.9 (3)
C9A—C5A—N5	120.0 (2)	C22—C23—N23	117.6 (3)
C7—C6—C5A	121.1 (3)	C25—C24—C23	116.1 (2)
C7—C6—H6	119.4	C25—C24—H24	122.0
C5A—C6—H6	119.4	C23—C24—H24	122.0
C8—C7—C6	118.6 (3)	C24—C25—C26	123.0 (3)
C8—C7—H7	120.7	C24—C25—N25	118.4 (3)
C6—C7—H7	120.7	C26—C25—N25	118.5 (3)
C7—C8—C9	121.7 (3)	C25—C26—C21	119.1 (2)
C7—C8—Cl8	118.3 (2)	C25—C26—H26	120.4
C9—C8—Cl8	120.0 (2)	C21—C26—H26	120.4
C8—C9—C9A	120.3 (3)	O21—C27—O22	126.7 (2)
C8—C9—H9	119.9	O21—C27—C21	118.2 (2)
C9A—C9—H9	119.9	O22—C27—C21	115.0 (2)
C9—C9A—N10	117.8 (2)	O24—N23—O23	124.6 (3)
C9—C9A—C5A	118.1 (2)	O24—N23—C23	117.1 (3)
N10—C9A—C5A	123.6 (2)	O23—N23—C23	118.3 (3)
C11—N10—C9A	122.8 (2)	O26—N25—O25	124.3 (3)
N10—C11—N11	118.1 (2)	O26—N25—C25	118.2 (3)
N10—C11—C11A	125.2 (2)	O25—N25—C25	117.6 (3)
N11—C11—C11A	116.6 (2)	O31—S31—C32	104.5 (2)
C1—C11A—C4A	118.7 (2)	O31—S31—C31	107.6 (3)
C1—C11A—C11	121.9 (2)	C32—S31—C31	99.1 (4)
C4A—C11A—C11	119.5 (2)	S31—C31—H31A	109.5
C11—N11—C16	126.01 (19)	S31—C31—H31B	109.5
C11—N11—C12	120.80 (19)	H31A—C31—H31B	109.5
C16—N11—C12	112.91 (18)	S31—C31—H31C	109.5
N11—C12—C13	111.7 (2)	H31A—C31—H31C	109.5
N11—C12—H12A	109.3	H31B—C31—H31C	109.5
C13—C12—H12A	109.3	S31—C32—H32A	109.5
N11—C12—H12B	109.3	S31—C32—H32B	109.5
C13—C12—H12B	109.3	H32A—C32—H32B	109.5
H12A—C12—H12B	107.9	S31—C32—H32C	109.5
N14—C13—C12	109.72 (19)	H32A—C32—H32C	109.5
N14—C13—H13A	109.7	H32B—C32—H32C	109.5
C12—C13—H13A	109.7	O41—S41—C42	106.5 (4)
N14—C13—H13B	109.7	O41—S41—C41	108.6 (5)
C12—C13—H13B	109.7	C42—S41—C41	99.8 (5)
H13A—C13—H13B	108.2	S41—C41—H41A	109.5
C17—N14—C15	112.1 (2)	S41—C41—H41B	109.5
C17—N14—C13	112.7 (2)	H41A—C41—H41B	109.5
C15—N14—C13	109.17 (18)	S41—C41—H41C	109.5
C17—N14—H14	104.9 (16)	H41A—C41—H41C	109.5
C15—N14—H14	110.5 (16)	H41B—C41—H41C	109.5
C13—N14—H14	107.3 (16)	S41—C42—H42A	109.5
N14—C15—C16	110.49 (19)	S41—C42—H42B	109.5
N14—C15—H15A	109.6	H42A—C42—H42B	109.5
C16—C15—H15A	109.6	S41—C42—H42C	109.5
N14—C15—H15B	109.6	H42A—C42—H42C	109.5
C16—C15—H15B	109.6	H42B—C42—H42C	109.5
H15A—C15—H15B	108.1		
			
C11A—C1—C2—C3	1.1 (4)	C11A—C11—N11—C16	16.8 (3)
C1—C2—C3—C4	−5.1 (4)	N10—C11—N11—C12	6.3 (3)
C2—C3—C4—C4A	3.6 (4)	C11A—C11—N11—C12	−169.7 (2)
C3—C4—C4A—C11A	1.8 (4)	C11—N11—C12—C13	−120.8 (2)
C3—C4—C4A—N5	−178.5 (2)	C16—N11—C12—C13	53.4 (3)
C4—C4A—N5—C5A	115.1 (3)	N11—C12—C13—N14	−56.7 (3)
C11A—C4A—N5—C5A	−65.2 (3)	C12—C13—N14—C17	−175.3 (2)
C4A—N5—C5A—C6	−117.8 (2)	C12—C13—N14—C15	59.5 (2)
C4A—N5—C5A—C9A	63.5 (3)	C17—N14—C15—C16	175.4 (2)
C9A—C5A—C6—C7	−0.1 (4)	C13—N14—C15—C16	−59.1 (2)
N5—C5A—C6—C7	−178.8 (2)	C11—N11—C16—C15	121.5 (2)
C5A—C6—C7—C8	−1.9 (4)	C12—N11—C16—C15	−52.4 (3)
C6—C7—C8—C9	2.3 (4)	N14—C15—C16—N11	55.3 (3)
C6—C7—C8—Cl8	−177.8 (2)	C26—C21—C22—C23	−0.1 (3)
C7—C8—C9—C9A	−0.8 (4)	C27—C21—C22—C23	−177.5 (2)
Cl8—C8—C9—C9A	179.38 (18)	C21—C22—C23—C24	−0.4 (4)
C8—C9—C9A—N10	171.3 (2)	C21—C22—C23—N23	176.6 (2)
C8—C9—C9A—C5A	−1.3 (3)	C22—C23—C24—C25	0.3 (4)
C6—C5A—C9A—C9	1.7 (3)	N23—C23—C24—C25	−176.7 (2)
N5—C5A—C9A—C9	−179.6 (2)	C23—C24—C25—C26	0.3 (4)
C6—C5A—C9A—N10	−170.4 (2)	C23—C24—C25—N25	177.4 (2)
N5—C5A—C9A—N10	8.2 (3)	C24—C25—C26—C21	−0.7 (3)
C9—C9A—N10—C11	146.3 (2)	N25—C25—C26—C21	−177.8 (2)
C5A—C9A—N10—C11	−41.5 (3)	C22—C21—C26—C25	0.6 (3)
C9A—N10—C11—N11	176.4 (2)	C27—C21—C26—C25	178.1 (2)
C9A—N10—C11—C11A	−7.9 (4)	C22—C21—C27—O21	177.5 (2)
C2—C1—C11A—C4A	4.3 (3)	C26—C21—C27—O21	0.0 (3)
C2—C1—C11A—C11	−177.0 (2)	C22—C21—C27—O22	−2.0 (3)
C4—C4A—C11A—C1	−5.7 (3)	C26—C21—C27—O22	−179.5 (2)
N5—C4A—C11A—C1	174.6 (2)	C24—C23—N23—O24	−0.8 (4)
C4—C4A—C11A—C11	175.5 (2)	C22—C23—N23—O24	−177.9 (3)
N5—C4A—C11A—C11	−4.1 (3)	C24—C23—N23—O23	177.6 (3)
N10—C11—C11A—C1	−129.2 (3)	C22—C23—N23—O23	0.5 (4)
N11—C11—C11A—C1	46.5 (3)	C24—C25—N25—O26	−178.3 (2)
N10—C11—C11A—C4A	49.5 (3)	C26—C25—N25—O26	−1.1 (4)
N11—C11—C11A—C4A	−134.7 (2)	C24—C25—N25—O25	1.6 (4)
N10—C11—N11—C16	−167.1 (2)	C26—C25—N25—O25	178.8 (3)

### (I) Clozapinium 3,5-dinitrobenzoate dimethyl sulfoxide monosolvate Hydrogen-bond geometry (Å, º)

**Table:**

*D*—H···*A*	*D*—H	H···*A*	*D*···*A*	*D*—H···*A*
N5—H5···O31	0.83 (3)	2.10 (3)	2.921 (4)	170 (3)
N14—H14···O22	1.00 (3)	1.58 (3)	2.575 (3)	176 (2)
C4—H4···O31	0.95	2.58	3.342 (4)	137
C4—H4···O41	0.95	2.38	3.208 (7)	146
C6—H6···O31	0.95	2.54	3.313 (5)	139

### (II) Clozapinium hydrogen maleate 0.21-hydrate Crystal data

**Table:**

C_18_H_20_ClN_4_^+^·C_4_H_3_O_4_^−^·0.21H_2_O	*F*(000) = 936.4
*M**_r_* = 446.68	*D*_x_ = 1.335 Mg m^−^^3^
Monoclinic, *P*2_1_/*c*	Cu *K*α radiation, λ = 1.54184 Å
*a* = 9.7166 (3) Å	Cell parameters from 4228 reflections
*b* = 9.9699 (2) Å	θ = 3.9–71.2°
*c* = 23.1059 (6) Å	µ = 1.84 mm^−^^1^
β = 96.800 (3)°	*T* = 173 K
*V* = 2222.60 (10) Å^3^	Block, colourless
*Z* = 4	0.46 × 0.32 × 0.22 mm

### (II) Clozapinium hydrogen maleate 0.21-hydrate Data collection

**Table:**

Agilent Eos Gemini diffractometer	3552 reflections with *I* > 2σ(*I*)
Radiation source: Enhance (Cu) X-ray Source	*R*_int_ = 0.026
ω scans	θ_max_ = 71.2°, θ_min_ = 3.9°
Absorption correction: multi-scan (CrysAlis RED; Agilent, 2012)	*h* = −11→11
*T*_min_ = 0.440, *T*_max_ = 0.668	*k* = −12→7
8634 measured reflections	*l* = −28→27
4228 independent reflections	

### (II) Clozapinium hydrogen maleate 0.21-hydrate Refinement

**Table:**

Refinement on *F*^2^	0 restraints
Least-squares matrix: full	Hydrogen site location: difference Fourier map
*R*[*F*^2^ > 2σ(*F*^2^)] = 0.048	H atoms treated by a mixture of independent and constrained refinement
*wR*(*F*^2^) = 0.133	*w* = 1/[σ^2^(*F*_o_^2^) + (0.069*P*)^2^ + 0.8133*P*] where *P* = (*F*_o_^2^ + 2*F*_c_^2^)/3
*S* = 1.03	(Δ/σ)_max_ < 0.001
4228 reflections	Δρ_max_ = 0.46 e Å^−^^3^
295 parameters	Δρ_min_ = −0.30 e Å^−^^3^

### (II) Clozapinium hydrogen maleate 0.21-hydrate Special details

**Table:**

Geometry. All e.s.d.'s (except the e.s.d. in the dihedral angle between two l.s. planes) are estimated using the full covariance matrix. The cell e.s.d.'s are taken into account individually in the estimation of e.s.d.'s in distances, angles and torsion angles; correlations between e.s.d.'s in cell parameters are only used when they are defined by crystal symmetry. An approximate (isotropic) treatment of cell e.s.d.'s is used for estimating e.s.d.'s involving l.s. planes.

### (II) Clozapinium hydrogen maleate 0.21-hydrate Fractional atomic coordinates and isotropic or equivalent isotropic displacement parameters (Å^2^)

**Table:**

	*x*	*y*	*z*	*U*_iso_*/*U*_eq_	Occ. (<1)
C1	0.6656 (2)	0.4997 (2)	0.23723 (9)	0.0361 (4)	
H1	0.7048	0.4466	0.2691	0.043*	
C2	0.7438 (2)	0.5303 (2)	0.19279 (9)	0.0417 (5)	
H2	0.8362	0.4981	0.1940	0.050*	
C3	0.6866 (2)	0.6083 (2)	0.14640 (9)	0.0409 (5)	
H3	0.7417	0.6340	0.1169	0.049*	
C4	0.5503 (2)	0.6488 (2)	0.14272 (8)	0.0356 (4)	
H4	0.5110	0.6994	0.1100	0.043*	
C4A	0.46942 (19)	0.61605 (19)	0.18684 (8)	0.0291 (4)	
N5	0.32745 (17)	0.65240 (17)	0.18232 (7)	0.0326 (3)	
H5	0.307 (2)	0.703 (3)	0.1527 (11)	0.039*	
C5A	0.23552 (18)	0.54101 (19)	0.18333 (8)	0.0296 (4)	
C6	0.1401 (2)	0.5111 (2)	0.13518 (8)	0.0357 (4)	
H6	0.1364	0.5660	0.1014	0.043*	
C7	0.0501 (2)	0.4028 (2)	0.13556 (9)	0.0385 (5)	
H7	−0.0150	0.3835	0.1026	0.046*	
C8	0.05780 (19)	0.3239 (2)	0.18497 (9)	0.0360 (4)	
Cl8	−0.05340 (6)	0.18656 (6)	0.18694 (3)	0.0560 (2)	
C9	0.14961 (19)	0.3526 (2)	0.23364 (9)	0.0334 (4)	
H9	0.1507	0.2984	0.2675	0.040*	
C9A	0.24090 (18)	0.46068 (19)	0.23353 (8)	0.0290 (4)	
N10	0.32420 (16)	0.48876 (16)	0.28580 (6)	0.0301 (3)	
C11	0.45129 (18)	0.52545 (18)	0.28708 (8)	0.0283 (4)	
C11A	0.52924 (19)	0.54587 (19)	0.23581 (8)	0.0293 (4)	
N11	0.52983 (16)	0.53872 (17)	0.34067 (6)	0.0315 (4)	
C12	0.46621 (19)	0.50463 (19)	0.39301 (7)	0.0300 (4)	
H12A	0.5381	0.4687	0.4229	0.036*	
H12B	0.3960	0.4336	0.3834	0.036*	
C13	0.39818 (18)	0.62443 (19)	0.41749 (8)	0.0302 (4)	
H13A	0.3214	0.6569	0.3889	0.036*	
H13B	0.3592	0.5986	0.4536	0.036*	
N14	0.50291 (17)	0.73320 (17)	0.43053 (7)	0.0324 (4)	
H14	0.565 (3)	0.702 (2)	0.4574 (11)	0.039*	
C15	0.5702 (2)	0.7683 (2)	0.37746 (8)	0.0382 (5)	
H15A	0.6437	0.8358	0.3878	0.046*	
H15B	0.5004	0.8082	0.3477	0.046*	
C16	0.63243 (19)	0.6451 (2)	0.35254 (8)	0.0361 (4)	
H16A	0.6701	0.6694	0.3160	0.043*	
H16B	0.7101	0.6120	0.3805	0.043*	
C17	0.4417 (3)	0.8518 (2)	0.45611 (11)	0.0561 (6)	
H17A	0.3690	0.8896	0.4278	0.084*	
H17B	0.5140	0.9193	0.4661	0.084*	
H17C	0.4017	0.8251	0.4914	0.084*	
C21	1.1468 (2)	0.6834 (2)	0.54450 (9)	0.0415 (5)	
O21	1.1086 (2)	0.7228 (3)	0.49282 (8)	0.0910 (9)	
H21	1.006 (5)	0.739 (5)	0.488 (2)	0.137*	
O22	1.26642 (15)	0.69322 (19)	0.56647 (7)	0.0533 (4)	
C22	1.0433 (2)	0.6232 (3)	0.57919 (10)	0.0490 (6)	
H22	1.0817	0.5844	0.6151	0.059*	
C23	0.9056 (2)	0.6141 (3)	0.56863 (10)	0.0494 (6)	
H23	0.8618	0.5719	0.5984	0.059*	
C24	0.8103 (2)	0.6601 (2)	0.51734 (8)	0.0356 (4)	
O23	0.68470 (15)	0.6486 (2)	0.51985 (7)	0.0526 (4)	
O24	0.85884 (18)	0.7068 (3)	0.47375 (8)	0.0810 (8)	
O31	0.7719 (11)	0.9745 (11)	0.5046 (5)	0.074 (4)*	0.210 (7)
H31A	0.8183	0.9016	0.4951	0.111*	0.210 (7)
H31B	0.8247	1.0490	0.5081	0.111*	0.210 (7)

### (II) Clozapinium hydrogen maleate 0.21-hydrate Atomic displacement parameters (Å^2^)

**Table:**

	*U*^11^	*U*^22^	*U*^33^	*U*^12^	*U*^13^	*U*^23^
C1	0.0326 (9)	0.0429 (11)	0.0324 (9)	0.0035 (8)	0.0016 (7)	−0.0027 (8)
C2	0.0333 (10)	0.0513 (13)	0.0421 (11)	0.0024 (9)	0.0106 (8)	−0.0098 (10)
C3	0.0439 (11)	0.0483 (12)	0.0328 (10)	−0.0098 (9)	0.0148 (8)	−0.0075 (9)
C4	0.0435 (11)	0.0355 (10)	0.0273 (9)	−0.0063 (8)	0.0031 (8)	−0.0016 (8)
C4A	0.0319 (9)	0.0278 (9)	0.0271 (8)	−0.0028 (7)	0.0018 (7)	−0.0056 (7)
N5	0.0350 (8)	0.0308 (8)	0.0308 (8)	0.0020 (7)	−0.0011 (6)	0.0024 (7)
C5A	0.0259 (8)	0.0304 (9)	0.0321 (9)	0.0043 (7)	0.0013 (7)	−0.0053 (7)
C6	0.0331 (9)	0.0416 (11)	0.0310 (9)	0.0058 (8)	−0.0016 (7)	−0.0024 (8)
C7	0.0277 (9)	0.0469 (12)	0.0388 (10)	0.0021 (8)	−0.0047 (8)	−0.0118 (9)
C8	0.0250 (9)	0.0357 (10)	0.0467 (11)	−0.0019 (8)	0.0017 (8)	−0.0088 (9)
Cl8	0.0448 (3)	0.0505 (3)	0.0690 (4)	−0.0191 (2)	−0.0089 (3)	−0.0033 (3)
C9	0.0279 (9)	0.0355 (10)	0.0365 (9)	0.0002 (8)	0.0022 (7)	−0.0017 (8)
C9A	0.0245 (8)	0.0322 (9)	0.0301 (9)	0.0035 (7)	0.0020 (7)	−0.0059 (7)
N10	0.0299 (8)	0.0341 (8)	0.0261 (7)	−0.0022 (6)	0.0020 (6)	−0.0023 (6)
C11	0.0294 (9)	0.0293 (9)	0.0258 (8)	0.0004 (7)	0.0016 (7)	−0.0011 (7)
C11A	0.0297 (9)	0.0325 (9)	0.0254 (8)	−0.0012 (7)	0.0028 (7)	−0.0038 (7)
N11	0.0308 (8)	0.0407 (9)	0.0227 (7)	−0.0074 (7)	0.0025 (6)	0.0006 (6)
C12	0.0341 (9)	0.0316 (9)	0.0237 (8)	−0.0051 (7)	0.0012 (7)	0.0029 (7)
C13	0.0274 (8)	0.0358 (10)	0.0268 (8)	−0.0034 (7)	0.0001 (7)	0.0030 (7)
N14	0.0388 (9)	0.0302 (8)	0.0265 (7)	−0.0031 (7)	−0.0026 (6)	0.0028 (6)
C15	0.0458 (11)	0.0390 (11)	0.0281 (9)	−0.0157 (9)	−0.0022 (8)	0.0080 (8)
C16	0.0297 (9)	0.0537 (12)	0.0241 (8)	−0.0129 (9)	−0.0004 (7)	0.0012 (8)
C17	0.0829 (18)	0.0339 (11)	0.0526 (14)	0.0015 (12)	0.0122 (13)	−0.0047 (10)
C21	0.0347 (10)	0.0498 (12)	0.0383 (10)	−0.0006 (9)	−0.0032 (8)	−0.0123 (9)
O21	0.0376 (9)	0.183 (3)	0.0495 (10)	−0.0273 (13)	−0.0053 (8)	0.0325 (14)
O22	0.0315 (8)	0.0719 (12)	0.0541 (9)	0.0031 (7)	−0.0052 (7)	−0.0144 (8)
C22	0.0418 (11)	0.0594 (15)	0.0421 (11)	0.0017 (10)	−0.0102 (9)	0.0144 (10)
C23	0.0419 (11)	0.0644 (15)	0.0401 (11)	−0.0090 (11)	−0.0029 (9)	0.0215 (11)
C24	0.0354 (10)	0.0418 (11)	0.0278 (9)	−0.0107 (9)	−0.0041 (7)	0.0051 (8)
O23	0.0344 (8)	0.0847 (13)	0.0367 (8)	−0.0113 (8)	−0.0040 (6)	0.0215 (8)
O24	0.0400 (9)	0.158 (2)	0.0418 (9)	−0.0252 (11)	−0.0089 (7)	0.0419 (12)

### (II) Clozapinium hydrogen maleate 0.21-hydrate Geometric parameters (Å, º)

**Table:**

C1—C2	1.382 (3)	C12—C13	1.507 (3)
C1—C11A	1.399 (3)	C12—H12A	0.9900
C1—H1	0.9500	C12—H12B	0.9900
C2—C3	1.387 (3)	C13—N14	1.493 (2)
C2—H2	0.9500	C13—H13A	0.9900
C3—C4	1.377 (3)	C13—H13B	0.9900
C3—H3	0.9500	N14—C17	1.478 (3)
C4—C4A	1.397 (3)	N14—C15	1.498 (2)
C4—H4	0.9500	N14—H14	0.87 (3)
C4A—C11A	1.397 (3)	C15—C16	1.513 (3)
C4A—N5	1.418 (2)	C15—H15A	0.9900
N5—C5A	1.427 (3)	C15—H15B	0.9900
N5—H5	0.86 (3)	C16—H16A	0.9900
C5A—C6	1.394 (3)	C16—H16B	0.9900
C5A—C9A	1.405 (3)	C17—H17A	0.9800
C6—C7	1.390 (3)	C17—H17B	0.9800
C6—H6	0.9500	C17—H17C	0.9800
C7—C8	1.381 (3)	C21—O22	1.216 (3)
C7—H7	0.9500	C21—O21	1.270 (3)
C8—C9	1.380 (3)	C21—C22	1.485 (3)
C8—Cl8	1.748 (2)	O21—H21	1.00 (5)
C9—C9A	1.396 (3)	C22—C23	1.334 (3)
C9—H9	0.9500	C22—H22	0.9500
C9A—N10	1.400 (2)	C23—C24	1.487 (3)
N10—C11	1.285 (2)	C23—H23	0.9500
C11—N11	1.382 (2)	C24—O23	1.234 (2)
C11—C11A	1.494 (2)	C24—O24	1.251 (3)
N11—C16	1.459 (2)	O31—H31A	0.8959
N11—C12	1.462 (2)	O31—H31B	0.9007
			
C2—C1—C11A	120.84 (19)	N11—C12—H12B	109.2
C2—C1—H1	119.6	C13—C12—H12B	109.2
C11A—C1—H1	119.6	H12A—C12—H12B	107.9
C1—C2—C3	119.49 (19)	N14—C13—C12	109.42 (15)
C1—C2—H2	120.3	N14—C13—H13A	109.8
C3—C2—H2	120.3	C12—C13—H13A	109.8
C4—C3—C2	120.47 (18)	N14—C13—H13B	109.8
C4—C3—H3	119.8	C12—C13—H13B	109.8
C2—C3—H3	119.8	H13A—C13—H13B	108.2
C3—C4—C4A	120.39 (19)	C17—N14—C13	111.41 (17)
C3—C4—H4	119.8	C17—N14—C15	112.05 (17)
C4A—C4—H4	119.8	C13—N14—C15	110.95 (14)
C11A—C4A—C4	119.47 (17)	C17—N14—H14	106.2 (16)
C11A—C4A—N5	119.70 (16)	C13—N14—H14	106.3 (16)
C4—C4A—N5	120.83 (17)	C15—N14—H14	109.6 (16)
C4A—N5—C5A	113.90 (15)	N14—C15—C16	110.77 (16)
C4A—N5—H5	110.1 (16)	N14—C15—H15A	109.5
C5A—N5—H5	112.8 (17)	C16—C15—H15A	109.5
C6—C5A—C9A	119.55 (18)	N14—C15—H15B	109.5
C6—C5A—N5	120.79 (18)	C16—C15—H15B	109.5
C9A—C5A—N5	119.66 (16)	H15A—C15—H15B	108.1
C7—C6—C5A	121.36 (19)	N11—C16—C15	111.51 (16)
C7—C6—H6	119.3	N11—C16—H16A	109.3
C5A—C6—H6	119.3	C15—C16—H16A	109.3
C8—C7—C6	118.39 (18)	N11—C16—H16B	109.3
C8—C7—H7	120.8	C15—C16—H16B	109.3
C6—C7—H7	120.8	H16A—C16—H16B	108.0
C9—C8—C7	121.44 (19)	N14—C17—H17A	109.5
C9—C8—Cl8	118.90 (17)	N14—C17—H17B	109.5
C7—C8—Cl8	119.65 (15)	H17A—C17—H17B	109.5
C8—C9—C9A	120.55 (19)	N14—C17—H17C	109.5
C8—C9—H9	119.7	H17A—C17—H17C	109.5
C9A—C9—H9	119.7	H17B—C17—H17C	109.5
C9—C9A—N10	117.09 (17)	O22—C21—O21	121.8 (2)
C9—C9A—C5A	118.67 (17)	O22—C21—C22	118.8 (2)
N10—C9A—C5A	124.00 (17)	O21—C21—C22	119.42 (19)
C11—N10—C9A	122.17 (15)	C21—O21—H21	109 (3)
N10—C11—N11	118.36 (16)	C23—C22—C21	131.1 (2)
N10—C11—C11A	126.65 (16)	C23—C22—H22	114.5
N11—C11—C11A	114.80 (15)	C21—C22—H22	114.5
C4A—C11A—C1	119.06 (17)	C22—C23—C24	129.9 (2)
C4A—C11A—C11	120.61 (16)	C22—C23—H23	115.1
C1—C11A—C11	120.28 (17)	C24—C23—H23	115.1
C11—N11—C16	122.04 (15)	O23—C24—O24	122.78 (19)
C11—N11—C12	118.45 (15)	O23—C24—C23	117.38 (18)
C16—N11—C12	111.09 (14)	O24—C24—C23	119.84 (19)
N11—C12—C13	111.93 (15)	C24—O24—H21	110.7 (19)
N11—C12—H12A	109.2	H31A—O31—H31B	113.2
C13—C12—H12A	109.2		
			
C11A—C1—C2—C3	0.3 (3)	C4—C4A—C11A—C11	171.79 (17)
C1—C2—C3—C4	−3.7 (3)	N5—C4A—C11A—C11	−8.6 (3)
C2—C3—C4—C4A	2.4 (3)	C2—C1—C11A—C4A	4.3 (3)
C3—C4—C4A—C11A	2.3 (3)	C2—C1—C11A—C11	−173.05 (19)
C3—C4—C4A—N5	−177.34 (18)	N10—C11—C11A—C4A	45.2 (3)
C11A—C4A—N5—C5A	−59.5 (2)	N11—C11—C11A—C4A	−139.81 (18)
C4—C4A—N5—C5A	120.07 (19)	N10—C11—C11A—C1	−137.5 (2)
C4A—N5—C5A—C6	−116.17 (19)	N11—C11—C11A—C1	37.5 (3)
C4A—N5—C5A—C9A	64.0 (2)	N10—C11—N11—C16	−142.60 (19)
C9A—C5A—C6—C7	−0.3 (3)	C11A—C11—N11—C16	42.0 (2)
N5—C5A—C6—C7	179.87 (17)	N10—C11—N11—C12	2.7 (3)
C5A—C6—C7—C8	−0.4 (3)	C11A—C11—N11—C12	−172.74 (16)
C6—C7—C8—C9	1.6 (3)	C11—N11—C12—C13	−91.0 (2)
C6—C7—C8—Cl8	−179.85 (15)	C16—N11—C12—C13	57.8 (2)
C7—C8—C9—C9A	−2.0 (3)	N11—C12—C13—N14	−57.45 (19)
Cl8—C8—C9—C9A	179.39 (14)	C12—C13—N14—C17	−178.53 (16)
C8—C9—C9A—N10	175.80 (17)	C12—C13—N14—C15	55.9 (2)
C8—C9—C9A—C5A	1.2 (3)	C17—N14—C15—C16	179.67 (18)
C6—C5A—C9A—C9	−0.1 (3)	C13—N14—C15—C16	−55.1 (2)
N5—C5A—C9A—C9	179.71 (16)	C11—N11—C16—C15	91.5 (2)
C6—C5A—C9A—N10	−174.23 (16)	C12—N11—C16—C15	−56.0 (2)
N5—C5A—C9A—N10	5.6 (3)	N14—C15—C16—N11	54.9 (2)
C9—C9A—N10—C11	141.11 (19)	O22—C21—C22—C23	171.5 (3)
C5A—C9A—N10—C11	−44.6 (3)	O21—C21—C22—C23	−8.7 (5)
C9A—N10—C11—N11	−173.90 (17)	C21—C22—C23—C24	1.3 (5)
C9A—N10—C11—C11A	0.9 (3)	C22—C23—C24—O23	−174.8 (3)
C4—C4A—C11A—C1	−5.6 (3)	C22—C23—C24—O24	6.0 (5)
N5—C4A—C11A—C1	174.05 (17)		

### (II) Clozapinium hydrogen maleate 0.21-hydrate Hydrogen-bond geometry (Å, º)

Cg1 and Cg2 represent the centroids of the rings (C5A,C6–C9,C9A) and (C1–C4,C4A,C11A), respectively.

**Table:**

*D*—H···*A*	*D*—H	H···*A*	*D*···*A*	*D*—H···*A*
N5—H5···O22^i^	0.86 (3)	2.24 (3)	3.084 (2)	170 (3)
N14—H14···O23	0.87 (3)	1.82 (3)	2.688 (2)	173 (2)
O21—H21···O24	1.00 (5)	1.46 (5)	2.420 (3)	157 (5)
O31—H31*A*···O24	0.90	2.05	2.913 (11)	160
O31—H31*B*···O21^ii^	0.90	2.37	3.232 (11)	161
C12—H12*A*···O22^iii^	0.99	2.48	3.308 (2)	141
C15—H15*A*···*Cg*1^iv^	0.99	2.95	3.642 (2)	128
C15—H15*B*···*Cg*2^iv^	0.99	2.95	3.759 (2)	139

Symmetry codes: (i) *x*−1, −*y*+3/2, *z*−1/2; (ii) −*x*+2, −*y*+2, −*z*+1; (iii) −*x*+2, −*y*+1, −*z*+1; (iv) −*x*+1, *y*+1/2, −*z*+1/2.

### (III) Clozapinium 2-hydroxybenzoate Crystal data

**Table:**

C_18_H_20_ClN_4_^+^·C_7_H_5_O_3_^−^	*F*(000) = 976
*M**_r_* = 464.94	*D*_x_ = 1.335 Mg m^−^^3^
Monoclinic, *C**c*	Cu *K*α radiation, λ = 1.54184 Å
*a* = 17.4296 (5) Å	Cell parameters from 4069 reflections
*b* = 15.3728 (5) Å	θ = 3.8–72.6°
*c* = 8.6359 (3) Å	µ = 1.75 mm^−^^1^
β = 90.325 (3)°	*T* = 173 K
*V* = 2313.88 (13) Å^3^	Block, colourless
*Z* = 4	0.42 × 0.36 × 0.20 mm

### (III) Clozapinium 2-hydroxybenzoate Data collection

**Table:**

Agilent Eos Gemini diffractometer	3962 reflections with *I* > 2σ(*I*)
Radiation source: Enhance (Cu) X-ray Source	*R*_int_ = 0.048
ω scans	θ_max_ = 72.6°, θ_min_ = 3.8°
Absorption correction: multi-scan (CrysAlis RED; Agilent, 2012)	*h* = −21→21
*T*_min_ = 0.399, *T*_max_ = 0.705	*k* = −10→18
7244 measured reflections	*l* = −10→10
4069 independent reflections	

### (III) Clozapinium 2-hydroxybenzoate Refinement

**Table:**

Refinement on *F*^2^	Hydrogen site location: difference Fourier map
Least-squares matrix: full	H atoms treated by a mixture of independent and constrained refinement
*R*[*F*^2^ > 2σ(*F*^2^)] = 0.056	*w* = 1/[σ^2^(*F*_o_^2^) + (0.1069*P*)^2^] where *P* = (*F*_o_^2^ + 2*F*_c_^2^)/3
*wR*(*F*^2^) = 0.141	(Δ/σ)_max_ < 0.001
*S* = 1.08	Δρ_max_ = 0.53 e Å^−^^3^
4069 reflections	Δρ_min_ = −0.36 e Å^−^^3^
308 parameters	Absolute structure: Flack *x* determined using 1674 quotients [(*I*^+^)-(*I*^-^)]/[(*I*^+^)+(*I*^-^)] (Parsons *et al.*, 2013)
2 restraints	Absolute structure parameter: −0.022 (17)

### (III) Clozapinium 2-hydroxybenzoate Special details

**Table:**

Geometry. All e.s.d.'s (except the e.s.d. in the dihedral angle between two l.s. planes) are estimated using the full covariance matrix. The cell e.s.d.'s are taken into account individually in the estimation of e.s.d.'s in distances, angles and torsion angles; correlations between e.s.d.'s in cell parameters are only used when they are defined by crystal symmetry. An approximate (isotropic) treatment of cell e.s.d.'s is used for estimating e.s.d.'s involving l.s. planes.

### (III) Clozapinium 2-hydroxybenzoate Fractional atomic coordinates and isotropic or equivalent isotropic displacement parameters (Å^2^)

**Table:**

	*x*	*y*	*z*	*U*_iso_*/*U*_eq_	
C1	0.68573 (19)	0.4925 (2)	0.8347 (4)	0.0296 (6)	
H1	0.6566	0.5324	0.8945	0.036*	
C2	0.7650 (2)	0.4966 (2)	0.8402 (4)	0.0346 (7)	
H2	0.7899	0.5394	0.9018	0.042*	
C3	0.80783 (19)	0.4372 (2)	0.7545 (4)	0.0338 (7)	
H3	0.8623	0.4405	0.7552	0.041*	
C4	0.77094 (19)	0.3730 (2)	0.6681 (4)	0.0322 (7)	
H4	0.8003	0.3310	0.6136	0.039*	
C4A	0.69150 (18)	0.3700 (2)	0.6611 (3)	0.0273 (6)	
N5	0.65422 (16)	0.30589 (18)	0.5690 (3)	0.0306 (6)	
H5	0.688 (3)	0.276 (3)	0.511 (6)	0.037*	
C5A	0.60999 (17)	0.2452 (2)	0.6565 (3)	0.0290 (6)	
C6	0.6279 (2)	0.1568 (2)	0.6588 (4)	0.0368 (7)	
H6	0.6702	0.1363	0.6004	0.044*	
C7	0.5850 (2)	0.0983 (2)	0.7448 (5)	0.0403 (8)	
H7	0.5969	0.0380	0.7442	0.048*	
C8	0.5249 (2)	0.1300 (2)	0.8312 (5)	0.0369 (7)	
Cl8	0.47247 (6)	0.05843 (6)	0.94789 (14)	0.0561 (3)	
C9	0.50474 (19)	0.2173 (2)	0.8300 (4)	0.0332 (7)	
H9	0.4622	0.2369	0.8886	0.040*	
C9A	0.54720 (18)	0.2761 (2)	0.7424 (4)	0.0287 (6)	
N10	0.51934 (16)	0.36170 (18)	0.7350 (3)	0.0294 (5)	
C11	0.56224 (18)	0.4295 (2)	0.7362 (3)	0.0267 (6)	
C11A	0.64754 (19)	0.4310 (2)	0.7428 (3)	0.0272 (6)	
N11	0.52619 (16)	0.50963 (18)	0.7496 (3)	0.0303 (6)	
C12	0.44338 (19)	0.5111 (2)	0.7757 (4)	0.0334 (7)	
H12A	0.4281	0.4591	0.8356	0.040*	
H12B	0.4159	0.5098	0.6751	0.040*	
C13	0.4218 (2)	0.5929 (2)	0.8641 (4)	0.0357 (7)	
H13A	0.3654	0.5949	0.8774	0.043*	
H13B	0.4458	0.5914	0.9682	0.043*	
N14	0.44753 (17)	0.6726 (2)	0.7804 (3)	0.0331 (6)	
H14	0.423 (3)	0.675 (3)	0.690 (7)	0.040*	
C15	0.5313 (2)	0.6687 (2)	0.7446 (4)	0.0338 (7)	
H15A	0.5614	0.6712	0.8422	0.041*	
H15B	0.5457	0.7195	0.6805	0.041*	
C16	0.55040 (19)	0.5851 (2)	0.6580 (4)	0.0306 (6)	
H16A	0.5237	0.5847	0.5565	0.037*	
H16B	0.6063	0.5821	0.6393	0.037*	
C17	0.4297 (3)	0.7522 (3)	0.8713 (5)	0.0482 (9)	
H17A	0.4588	0.7513	0.9687	0.072*	
H17B	0.3747	0.7539	0.8937	0.072*	
H17C	0.4439	0.8038	0.8114	0.072*	
C21	0.28195 (18)	0.6783 (2)	0.3237 (4)	0.0306 (6)	
C22	0.2327 (2)	0.6192 (3)	0.2489 (5)	0.0462 (9)	
C23	0.1947 (3)	0.6426 (4)	0.1142 (7)	0.0657 (14)	
H23A	0.1623	0.6020	0.0627	0.079*	
C24	0.2041 (3)	0.7258 (5)	0.0548 (6)	0.0673 (15)	
H24	0.1774	0.7422	−0.0369	0.081*	
C25	0.2518 (3)	0.7851 (3)	0.1272 (5)	0.0509 (10)	
H25	0.2576	0.8419	0.0856	0.061*	
C26	0.2911 (2)	0.7617 (2)	0.2602 (4)	0.0354 (7)	
H26	0.3245	0.8022	0.3089	0.042*	
C27	0.32403 (17)	0.6517 (2)	0.4680 (4)	0.0286 (6)	
O21	0.31483 (16)	0.57583 (18)	0.5174 (4)	0.0445 (6)	
O22	0.36650 (14)	0.70751 (16)	0.5327 (3)	0.0332 (5)	
O23	0.2212 (2)	0.5383 (2)	0.3064 (5)	0.0661 (10)	
H23	0.259 (6)	0.544 (6)	0.394 (13)	0.099*	

### (III) Clozapinium 2-hydroxybenzoate Atomic displacement parameters (Å^2^)

**Table:**

	*U*^11^	*U*^22^	*U*^33^	*U*^12^	*U*^13^	*U*^23^
C1	0.0314 (15)	0.0289 (13)	0.0285 (14)	0.0049 (11)	0.0008 (12)	−0.0026 (11)
C2	0.0345 (17)	0.0331 (15)	0.0363 (16)	−0.0012 (12)	−0.0036 (13)	−0.0015 (12)
C3	0.0239 (13)	0.0341 (15)	0.0433 (18)	0.0016 (11)	−0.0013 (12)	0.0033 (12)
C4	0.0311 (16)	0.0340 (15)	0.0317 (16)	0.0088 (12)	0.0028 (12)	0.0018 (12)
C4A	0.0305 (16)	0.0285 (14)	0.0229 (13)	0.0044 (11)	0.0013 (11)	0.0035 (10)
N5	0.0311 (13)	0.0348 (13)	0.0259 (12)	0.0058 (11)	0.0038 (10)	−0.0053 (10)
C5A	0.0255 (15)	0.0335 (15)	0.0279 (15)	0.0030 (11)	−0.0050 (12)	−0.0046 (11)
C6	0.0329 (15)	0.0346 (17)	0.0429 (19)	0.0062 (12)	−0.0030 (13)	−0.0084 (13)
C7	0.0354 (17)	0.0276 (15)	0.058 (2)	0.0028 (12)	−0.0092 (15)	−0.0047 (13)
C8	0.0300 (15)	0.0340 (16)	0.0466 (19)	−0.0048 (12)	−0.0060 (14)	0.0011 (13)
Cl8	0.0461 (5)	0.0410 (5)	0.0813 (8)	−0.0089 (4)	0.0056 (5)	0.0139 (4)
C9	0.0243 (14)	0.0387 (16)	0.0364 (17)	−0.0001 (11)	−0.0050 (12)	−0.0021 (12)
C9A	0.0241 (13)	0.0336 (15)	0.0281 (14)	0.0035 (11)	−0.0074 (11)	−0.0035 (11)
N10	0.0253 (11)	0.0345 (13)	0.0284 (13)	0.0068 (10)	−0.0013 (10)	0.0003 (10)
C11	0.0272 (15)	0.0338 (15)	0.0190 (13)	0.0063 (11)	0.0000 (10)	0.0004 (10)
C11A	0.0277 (15)	0.0296 (13)	0.0242 (14)	0.0034 (11)	0.0004 (11)	0.0022 (10)
N11	0.0272 (13)	0.0328 (13)	0.0310 (13)	0.0088 (10)	0.0028 (10)	0.0038 (10)
C12	0.0269 (15)	0.0355 (16)	0.0378 (17)	0.0087 (12)	0.0056 (12)	0.0048 (12)
C13	0.0338 (16)	0.0443 (18)	0.0290 (15)	0.0149 (14)	0.0048 (12)	0.0022 (13)
N14	0.0352 (14)	0.0367 (14)	0.0271 (14)	0.0137 (11)	−0.0073 (11)	−0.0033 (10)
C15	0.0329 (15)	0.0329 (15)	0.0357 (16)	0.0062 (12)	−0.0082 (13)	0.0000 (12)
C16	0.0315 (15)	0.0311 (15)	0.0292 (14)	0.0069 (11)	0.0017 (12)	0.0038 (12)
C17	0.053 (2)	0.049 (2)	0.0425 (19)	0.0227 (18)	−0.0116 (17)	−0.0150 (16)
C21	0.0250 (13)	0.0386 (16)	0.0282 (15)	0.0046 (12)	−0.0008 (11)	−0.0034 (12)
C22	0.0366 (18)	0.053 (2)	0.049 (2)	−0.0049 (16)	−0.0060 (16)	−0.0092 (17)
C23	0.053 (3)	0.093 (4)	0.051 (3)	−0.011 (3)	−0.021 (2)	−0.014 (3)
C24	0.052 (3)	0.114 (5)	0.036 (2)	0.012 (3)	−0.0204 (19)	0.002 (2)
C25	0.047 (2)	0.068 (3)	0.0374 (19)	0.0157 (19)	−0.0009 (17)	0.0149 (18)
C26	0.0311 (16)	0.0442 (18)	0.0307 (16)	0.0052 (13)	−0.0020 (13)	0.0025 (13)
C27	0.0212 (13)	0.0339 (15)	0.0308 (15)	0.0054 (11)	0.0030 (11)	−0.0010 (11)
O21	0.0373 (14)	0.0400 (13)	0.0562 (16)	−0.0004 (11)	−0.0038 (11)	0.0128 (12)
O22	0.0315 (11)	0.0375 (11)	0.0304 (11)	0.0034 (9)	−0.0075 (9)	−0.0002 (8)
O23	0.060 (2)	0.0520 (17)	0.086 (3)	−0.0213 (17)	−0.0142 (18)	−0.0066 (18)

### (III) Clozapinium 2-hydroxybenzoate Geometric parameters (Å, º)

**Table:**

C1—C2	1.383 (5)	C12—H12B	0.9900
C1—C11A	1.400 (5)	C13—N14	1.493 (5)
C1—H1	0.9500	C13—H13A	0.9900
C2—C3	1.395 (5)	C13—H13B	0.9900
C2—H2	0.9500	N14—C17	1.487 (4)
C3—C4	1.393 (5)	N14—C15	1.496 (4)
C3—H3	0.9500	N14—H14	0.88 (6)
C4—C4A	1.386 (4)	C15—C16	1.524 (4)
C4—H4	0.9500	C15—H15A	0.9900
C4A—C11A	1.404 (4)	C15—H15B	0.9900
C4A—N5	1.422 (4)	C16—H16A	0.9900
N5—C5A	1.429 (4)	C16—H16B	0.9900
N5—H5	0.90 (5)	C17—H17A	0.9800
C5A—C6	1.394 (5)	C17—H17B	0.9800
C5A—C9A	1.408 (4)	C17—H17C	0.9800
C6—C7	1.387 (6)	C21—C26	1.403 (5)
C6—H6	0.9500	C21—C22	1.406 (5)
C7—C8	1.379 (6)	C21—C27	1.499 (4)
C7—H7	0.9500	C22—O23	1.355 (6)
C8—C9	1.387 (5)	C22—C23	1.383 (7)
C8—Cl8	1.752 (4)	C23—C24	1.389 (9)
C9—C9A	1.394 (5)	C23—H23A	0.9500
C9—H9	0.9500	C24—C25	1.381 (9)
C9A—N10	1.405 (4)	C24—H24	0.9500
N10—C11	1.283 (5)	C25—C26	1.381 (5)
C11—N11	1.388 (4)	C25—H25	0.9500
C11—C11A	1.488 (4)	C26—H26	0.9500
N11—C12	1.462 (4)	C27—O21	1.253 (4)
N11—C16	1.467 (4)	C27—O22	1.261 (4)
C12—C13	1.520 (4)	O23—H23	1.00 (11)
C12—H12A	0.9900		
			
C2—C1—C11A	121.4 (3)	N14—C13—C12	111.1 (3)
C2—C1—H1	119.3	N14—C13—H13A	109.4
C11A—C1—H1	119.3	C12—C13—H13A	109.4
C1—C2—C3	119.3 (3)	N14—C13—H13B	109.4
C1—C2—H2	120.3	C12—C13—H13B	109.4
C3—C2—H2	120.3	H13A—C13—H13B	108.0
C4—C3—C2	120.1 (3)	C17—N14—C13	110.8 (3)
C4—C3—H3	120.0	C17—N14—C15	110.4 (3)
C2—C3—H3	120.0	C13—N14—C15	111.3 (3)
C4A—C4—C3	120.3 (3)	C17—N14—H14	110 (3)
C4A—C4—H4	119.9	C13—N14—H14	108 (3)
C3—C4—H4	119.9	C15—N14—H14	106 (3)
C4—C4A—C11A	120.3 (3)	N14—C15—C16	110.5 (3)
C4—C4A—N5	120.0 (3)	N14—C15—H15A	109.5
C11A—C4A—N5	119.7 (3)	C16—C15—H15A	109.5
C4A—N5—C5A	113.8 (2)	N14—C15—H15B	109.5
C4A—N5—H5	111 (3)	C16—C15—H15B	109.5
C5A—N5—H5	108 (3)	H15A—C15—H15B	108.1
C6—C5A—C9A	119.7 (3)	N11—C16—C15	109.8 (3)
C6—C5A—N5	121.5 (3)	N11—C16—H16A	109.7
C9A—C5A—N5	118.8 (3)	C15—C16—H16A	109.7
C7—C6—C5A	121.3 (3)	N11—C16—H16B	109.7
C7—C6—H6	119.4	C15—C16—H16B	109.7
C5A—C6—H6	119.4	H16A—C16—H16B	108.2
C8—C7—C6	118.3 (3)	N14—C17—H17A	109.5
C8—C7—H7	120.9	N14—C17—H17B	109.5
C6—C7—H7	120.9	H17A—C17—H17B	109.5
C7—C8—C9	122.1 (3)	N14—C17—H17C	109.5
C7—C8—Cl8	119.3 (3)	H17A—C17—H17C	109.5
C9—C8—Cl8	118.6 (3)	H17B—C17—H17C	109.5
C8—C9—C9A	119.7 (3)	C26—C21—C22	118.7 (3)
C8—C9—H9	120.1	C26—C21—C27	121.2 (3)
C9A—C9—H9	120.1	C22—C21—C27	120.0 (3)
C9—C9A—N10	116.6 (3)	O23—C22—C23	118.4 (4)
C9—C9A—C5A	119.0 (3)	O23—C22—C21	121.1 (4)
N10—C9A—C5A	124.1 (3)	C23—C22—C21	120.5 (4)
C11—N10—C9A	124.0 (3)	C22—C23—C24	119.6 (5)
N10—C11—N11	117.2 (3)	C22—C23—H23A	120.2
N10—C11—C11A	126.6 (3)	C24—C23—H23A	120.2
N11—C11—C11A	115.8 (3)	C25—C24—C23	120.9 (4)
C1—C11A—C4A	118.5 (3)	C25—C24—H24	119.6
C1—C11A—C11	120.3 (3)	C23—C24—H24	119.6
C4A—C11A—C11	121.2 (3)	C24—C25—C26	119.9 (5)
C11—N11—C12	118.3 (3)	C24—C25—H25	120.0
C11—N11—C16	121.7 (3)	C26—C25—H25	120.0
C12—N11—C16	111.0 (2)	C25—C26—C21	120.5 (4)
N11—C12—C13	109.7 (3)	C25—C26—H26	119.8
N11—C12—H12A	109.7	C21—C26—H26	119.8
C13—C12—H12A	109.7	O21—C27—O22	124.0 (3)
N11—C12—H12B	109.7	O21—C27—C21	118.3 (3)
C13—C12—H12B	109.7	O22—C27—C21	117.8 (3)
H12A—C12—H12B	108.2	C22—O23—H23	96 (5)
			
C11A—C1—C2—C3	−1.1 (5)	N11—C11—C11A—C1	32.9 (4)
C1—C2—C3—C4	−1.9 (5)	N10—C11—C11A—C4A	38.8 (4)
C2—C3—C4—C4A	2.8 (5)	N11—C11—C11A—C4A	−148.7 (3)
C3—C4—C4A—C11A	−0.8 (5)	N10—C11—N11—C12	5.5 (4)
C3—C4—C4A—N5	178.1 (3)	C11A—C11—N11—C12	−167.7 (3)
C4—C4A—N5—C5A	115.3 (3)	N10—C11—N11—C16	−138.6 (3)
C11A—C4A—N5—C5A	−65.7 (3)	C11A—C11—N11—C16	48.2 (4)
C4A—N5—C5A—C6	−117.1 (3)	C11—N11—C12—C13	151.8 (3)
C4A—N5—C5A—C9A	62.6 (4)	C16—N11—C12—C13	−60.6 (3)
C9A—C5A—C6—C7	−0.2 (5)	N11—C12—C13—N14	56.6 (4)
N5—C5A—C6—C7	179.6 (3)	C12—C13—N14—C17	−177.1 (3)
C5A—C6—C7—C8	−1.3 (5)	C12—C13—N14—C15	−53.8 (4)
C6—C7—C8—C9	2.3 (6)	C17—N14—C15—C16	177.3 (3)
C6—C7—C8—Cl8	−177.1 (3)	C13—N14—C15—C16	53.8 (4)
C7—C8—C9—C9A	−1.6 (5)	C11—N11—C16—C15	−152.7 (3)
Cl8—C8—C9—C9A	177.7 (3)	C12—N11—C16—C15	60.9 (4)
C8—C9—C9A—N10	174.2 (3)	N14—C15—C16—N11	−56.8 (4)
C8—C9—C9A—C5A	0.0 (5)	C26—C21—C22—O23	−179.2 (4)
C6—C5A—C9A—C9	0.8 (5)	C27—C21—C22—O23	1.2 (6)
N5—C5A—C9A—C9	−178.9 (3)	C26—C21—C22—C23	0.7 (6)
C6—C5A—C9A—N10	−172.9 (3)	C27—C21—C22—C23	−178.9 (4)
N5—C5A—C9A—N10	7.4 (5)	O23—C22—C23—C24	178.5 (5)
C9—C9A—N10—C11	141.9 (3)	C21—C22—C23—C24	−1.4 (8)
C5A—C9A—N10—C11	−44.2 (5)	C22—C23—C24—C25	0.9 (9)
C9A—N10—C11—N11	−171.4 (3)	C23—C24—C25—C26	0.3 (8)
C9A—N10—C11—C11A	1.0 (5)	C24—C25—C26—C21	−1.0 (6)
C2—C1—C11A—C4A	3.1 (5)	C22—C21—C26—C25	0.5 (5)
C2—C1—C11A—C11	−178.6 (3)	C27—C21—C26—C25	−180.0 (3)
C4—C4A—C11A—C1	−2.1 (4)	C26—C21—C27—O21	−178.5 (3)
N5—C4A—C11A—C1	179.0 (3)	C22—C21—C27—O21	1.1 (5)
C4—C4A—C11A—C11	179.6 (3)	C26—C21—C27—O22	1.7 (4)
N5—C4A—C11A—C11	0.6 (4)	C22—C21—C27—O22	−178.7 (3)
N10—C11—C11A—C1	−139.6 (3)		

### (III) Clozapinium 2-hydroxybenzoate Hydrogen-bond geometry (Å, º)

Cg1 represents the centroid of the ring (C5A,C6–C9,C9A).

**Table:**

*D*—H···*A*	*D*—H	H···*A*	*D*···*A*	*D*—H···*A*
N14—H14···O22	0.89 (6)	1.75 (6)	2.612 (4)	164 (5)
O23—H23···O21	1.01 (11)	1.52 (10)	2.507 (5)	166 (9)
C4—H4···O22^i^	0.95	2.33	3.261 (4)	166
C9—H9···O22^ii^	0.95	2.25	3.202 (4)	176
C12—H12*B*···O21	0.99	2.44	3.306 (5)	146
C15—H15*A*···N5^ii^	0.99	2.56	3.539 (4)	170
C24—H24···*Cg*1^iii^	0.95	2.83	3.637 (5)	144

Symmetry codes: (i) *x*+1/2, *y*−1/2, *z*; (ii) *x*, −*y*+1, *z*+1/2; (iii) *x*−1/2, *y*+1/2, *z*−1.
